# Directed Repeats Co-occur with Few Short-Dispersed Repeats in Plastid Genome of a Spikemoss, *Selaginella vardei* (Selaginellaceae, Lycopodiopsida)

**DOI:** 10.1186/s12864-019-5843-6

**Published:** 2019-06-11

**Authors:** Hong-Rui Zhang, Xian-Chun Zhang, Qiao-Ping Xiang

**Affiliations:** 1 State Key Laboratory of Systematic and Evolutionary Botany, Institute of Botany, the Chinese Academy of Sciences, Beijing, 100093 China; 2 University of Chinese Academy of Sciences, Beijing, 100049 China

**Keywords:** direct repeat, inversion, inverted repeat, plastome evolution, short dispersed repeats

## Abstract

**Background:**

It is hypothesized that the highly conserved inverted repeats (IR) structure of land plant plastid genomes (plastomes) is beneficial for stabilizing plastome organization, whereas the mechanism of the occurrence and stability maintenance of the recently reported direct repeats (DR) structure is yet awaiting further exploration. Here we describe the DR structure of the *Selaginella vardei* (Selaginellaceae) plastome, to elucidate the mechanism of DR occurrence and stability maintenance.

**Results:**

The plastome of *S. vardei* is 121,254 bp in length and encodes 76 genes, of which 62 encode proteins, 10 encode tRNAs, and four encode rRNAs. Unexpectedly, the two identical rRNA gene regions (13,893 bp) are arranged in a direct orientation (DR), rather than inverted. Comparing to the IR organization in *Isoetes flaccida* (Isoetaceae, Lycopodiopsida) plastome, a ca. 50-kb *trn*N-*trn*F inversion that spans one DR copy was found in the plastome of *S. vardei*, which might cause the orientation change. In addition, we find extremely rare short dispersed repeats (SDRs) in the plastomes of *S. vardei* and its closely related species *S. indica*.

**Conclusions:**

We suggest that the ca. 50-kb inversion resulted in the DR structure, and the reduction in SDRs plays a key role in maintaining the stability of plastomes with DR structure by avoiding potential secondary recombination. We further confirmed the presence of homologous recombination between DR regions, which are able to generate subgenomes and form diverse multimers. Our study deepens the understanding of *Selaginella* plastomes and provides new insights into the diverse plastome structures in land plants.

**Electronic supplementary material:**

The online version of this article (10.1186/s12864-019-5843-6) contains supplementary material, which is available to authorized users.

## Background

Chloroplasts in land plants have generally conserved genome structure, due to the constantly high selective pressures of photosynthesis [1]. Most plastomes are characterized by a quadripartite structure, which comprises two copies of an inverted repeat (IR) separating the large (LSC) and small (SSC) single copy regions [2]. The size of land plant plastomes usually ranges from 108 to 165 kb, and they generally contain 110–130 distinct genes including about 30 transfer RNA (tRNA) genes, four ribosomal RNA (rRNA) genes, and approximately 80 protein-coding genes involved in photosynthesis or other metabolic processes [2, 3]. The typical IR is usually 20–30 kb and the genes that form the core of the IR encode the ribosomal RNAs (23S, 16S, 5S, and 4.5S) [4]. While plastomes of most land plants possess the typical IR structure, several lineages of land plants only retain one copy of the IR, such as *Carnegiea gigantea* (Cactaceae) [5], *Erodium* (Geraniaceae) [6], and an IR-lacking clade of Fabaceae [7], as well as conifers [8], in which one IR has been either extremely shortened or completely lost.

The conserved IR structure across land plants is hypothesized to function in stabilizing the plastomes against major sequence rearrangements [9]. Palmer and Thompson [10] showed that rearrangement events were extremely rare in genomes with an IR, but increased remarkably in frequency when one IR copy was absent. Their hypothesis is supported by some recent studies using next generation sequencing. For example, *Trifolium* [11] and *Erodium texanum* [12] lack one copy of the IR and have highly rearranged plastomes. However, plastomes of *Pelargonium* [13] and *Trachelium* [14] are also highly rearranged, despite the presence of the IR structure, so that the existence of the IR appears to be insufficient to stabilize plastome structure [15]. A related hypothesis suggests that the incidence of short dispersed repeats (SDRs) is in fact more correlated with plastome instability [15]. Organellar DNA sequences of around 30 bp in length that may occur in direct or inverted forms are usually referred to as SDRs, and constitute, for example, more than 20% of the plastome of *Chlamydomonas reinhardtii* (green alga)[16]. Repeats larger than 1 kb can frequently recombine intra- or intermolecularly, and homologous recombination also occurs sporadically between short repeats larger than 50 bp [17]. Extensive studies have shown that genomes with massive rearrangement events tend to contain a high frequency of SDRs (e.g., *Trifolium* [11]; *Trachelium* [14]; *Pelargonium* [13]), whereas genomes containing virtually no SDRs have the conserved organization (*Erodium* [12]; algae [18, 19]).

Variation in number and orientation of rRNA-encoding repeats has been shown to be much more variable in algae than in land plants [20]. One to five copies of rRNA-encoding repeat are tandemly arranged in *Euglena* (green algae) [21], whereas in *Porphyra purpurea* (red alga), the two copies of the rRNA-encoding region are arranged into a direct repeat (DR) [22]. The mechanism of creating and maintaining this diversity remains unknown.

The lycophyte species *Selaginella tamariscina* and *S. kraussiana* have been recently reported to possess plastomes with two copies of rRNA-encoding repeat arranged into DR [23, 24]. The DR structure was proposed to have originated from the canonical IR via a large-scale inversion [24]. Here we confirmed plastomes with DR structure in *Selaginella* subg. *Rupestrae sensu* Weststrand and Korall [25]). We discovered that the two copies of DR were probably caused by a ca. 50-kb *trn*F-*trn*N inversion containing one DR copy. We also found SDRs to be rare in lycophyte plastomes with the DR organization in comparison to those with an IR. Considering the generation of subgenomes by the recombination between DR regions, we predict that the reduction of SDRs, co-occurring with the DR structure, plays an important role in maintaining the stability of plastome and surviving over long evolutionary history.

## Results

### The Unconventional Structure of the *S. vardei* Plastome

**Fig. 1:**
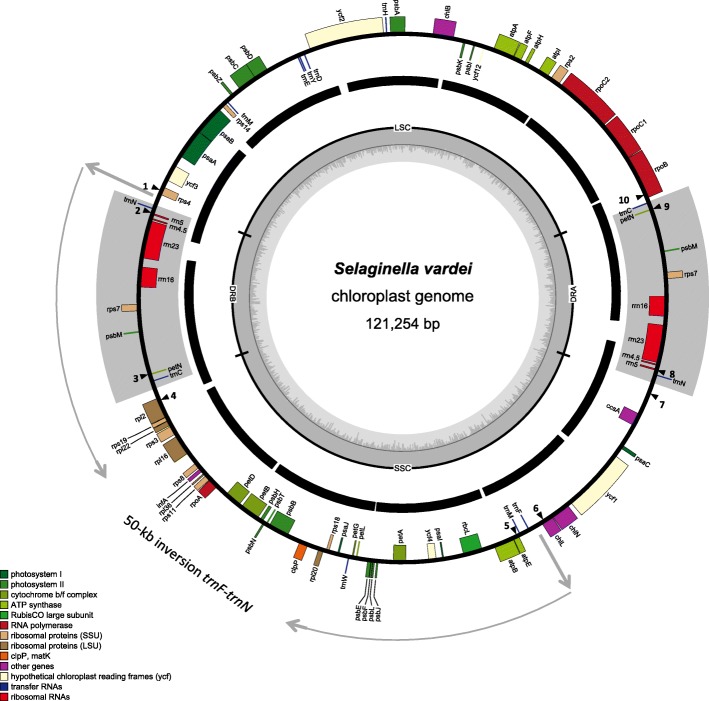
Unit-genome map of *S. vardei* shown as circular. The middle ring shows the mapping results of long-range PCR data with assembled sequence. The arrows point to the endpoints of the ca. 50-kb fragment (from *trn*N to *trn*F) inversion. The black triangular and numbers showed the position of primers for confirming the boundaries of DR regions and the 50-kb inversion for positive and negative controls

We sequenced the plastome of *S. vardei* (Fig. 1; MG272482) using Illumina HiSeq 2500 sequencing and assembled reads using de novo assembly methods. Coverage of the *S. vardei* plastome sequence is shown in Additional file 1: Figure S1c. The assembly result in Bandage 0.8.1 [26] was identical with the de novo methods, resulting in a circular plastome with the same organization (Additional file 1: Figure S1d). The plastome of *S. indica* (MK156801) was also sequenced and assembled; the organization and gene content are basically identical to that of *S. vardei*, therefore, we only described the detailed plastome characteristics of *S. vardei*.

The plastome of *S. vardei* is 121,254 bp long and contains 76 genes, including 62 protein-coding genes, four rRNA genes, and 10 tRNA genes (Additional file 6: Table S1). It is, therefore, much smaller than those of *S. uncinata* [27] and *S. moellendorffii* [28], apparently owing to gene losses of, for example, all the 11 *ndh* genes and two tRNA genes (*trn*Q and *trn*R). The plastome of *S. vardei* is similar to that of most land plants in having a quadripartite structure of two single-copy regions separated by two copies of a large rRNA-encoding repeat (13,893 bp). However, the two repeat copies were arranged into direct rather than inverted orientation, and the two single-copy regions are almost equal in size (47,676 bp and 45,792 bp, respectively).

### Confirmation of the Plastome Structure of *S. vardei*

The long PCR experiments across the whole plastome resulted in 12 products of the expected length (ca. 7–11 kb; Additional file 1: Figure S1a) using 12 pairs of primers (Additional file 7: Table S2) and the sequences were obtained using newly designed internal primers (Additional file 8: Table S3). We mapped the sequences of the long PCR products to the assembled plastome of *S. vardei* (Fig. 1). All the intergenic regions were covered by Sanger sequencing.

In addition, the amplification results of the DR structure and the 50-kb inversion, including six primer pairs for positive control and six for negative control, also supported the assembled plastome structure of *S. vardei* (Additional file 1: Figure S1b). The amplified fragments of positive control were consistent with the DR structure (1-2: *rps*4-*rrn*5 and 3-4: *pet*N-*rpl*2; 7-8: *ccs*A-*rrn*5 and 9-10: *pet*N-*rpo*B) and the ca. 50-kb inversion (1-2: *rps*4-*rrn*5 and 5-6: *atp*E-*chl*L) in *S. vardei*. Accordingly, the negative control consistent with amplification of a canonical IR structure (1-3: *rps4*-*pet*N and 2-4: *rrn*5-*rpl*2; 7-9: *ccs*A-*pet*N and 8-10: *rrn*5-*rpo*B) and without the ca. 50-kb inversion structure (1-5: *rps*4-*atp*E and 2-6: *rrn*5-*chl*L) yielded no PCR product. We further checked the sequences at boundaries of the DR region and the ca. 50-kb inversion. The assembled sequences from NGS were identical with sequences from Sanger sequencing. The congruent results from Sanger sequencing with those from our assembly strongly support the DR structure and the ca. 50-kb inversion in the *S. vardei* plastome.

### PCR Confirmation of DR Structure in Representatives of subg. *Rupestrae*

The PCR experiments on one additional individual of *S. vardei*, two individuals of *S. indica*, and one individual of *S. dregei* (all from *Selaginella* subg. *Rupestrae*; Table 1) yielded the expected products with consistent length (Additional file 2: Figure S2a). PCR products were consistent with DR structure and the ca. 50-kb inversion. The aligned sequences of the four samples showed several indels (Additional file 2: Figure S2b). Sequences in *S. dregei* is much more divergent in the number of indels and nucleotide sites when compared with *S. vardei* and *S. indica*. For example, a 475 bp deletion occurred at the region of *ccs*A-*rrn*5 in the *S. dregei* (collected from Kenya, Africa), which also exhibits a smaller PCR product than the other species (Additional file 2: Figure S2b: 26698, 7-8).

**Table 1:** Representatives related for confirmation of plastome structure of *S. vardei*

Species	Voucher	Locality
*S. vardei*	Zhang X. C. 6948 (PE)	Sichuan, China
*S. vardei*	Zhang X. C. et al. 836 (PE)	Tibet, China
*S. indica*	Zhang X. C. 5868 (PE)	Sichuan Province, China
*S. indica*	Zhang X. C. 6255 (PE)	Yunnan Province, China
*S. dregei*	Liu B. 26698 (PE)	Kenya

### Gene Order of Plastomes in *S. vardei* and Other Lycophytes

**Fig. 2:**
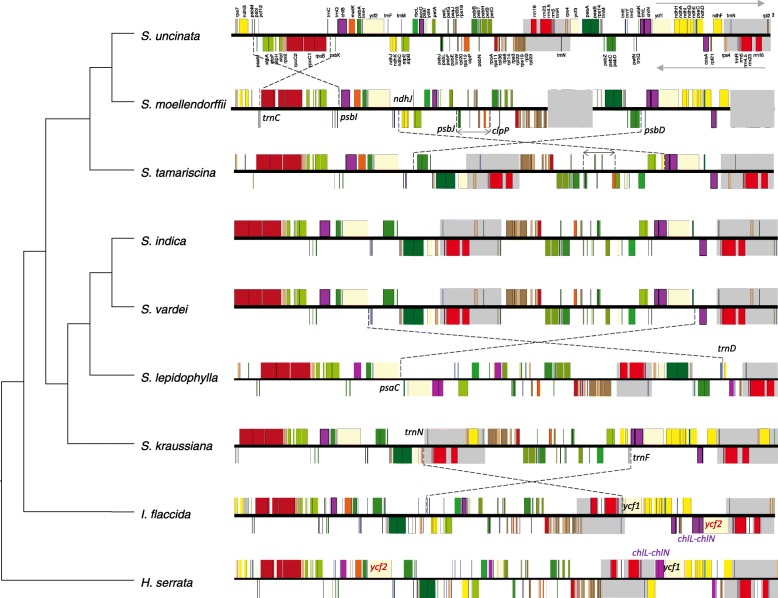
Linear maps of plastomes between *S. vardei* and other lycophytes showing the rearrangements. Upper arrow indicates genes transcribed in forward direction; lower arrow indicates genes transcribed in reverse direction. The red *ycf*2 indicates the translocation from LSC to SSC and the purple *chl*L-*chl*L indicates the inversion in *I. flaccida*

Dot plot analysis (Additional file 3: Figure S3) showed that plastomes of *Huperzia serrata* and *Isoetes flaccida* are basically syntenic, except the translocation of *ycf*2 and inversion of *chl*L-*chl*N in *I. flaccida* (Additional file 3: Figure S3a), whereas, plastomes of *S. vardei* were quite divergent from both of them (Additional file 3: Figure S3b). A large inversion was present in *S. vardei* that encompasses a ca. 50-kb region from *trn*N to *trn*F, its endpoints lying between *rps*4 and *trn*N at one end and between *trn*F and *chl*L at the other (Figs. 1 and 2). In *I. flaccida*, two tRNA genes, *trn*L-UAA and *trn*T-UGU were situated between *rps*4 and *trn*F in LSC region, whereas *trn*N was at the border of the IRb/SSC adjacent to *ycf*2. In *S. vardei*, however, *trn*L-UAA and *trn*T-UGU were absent, *trn*F was located in the SSC, and *trn*N was located at the border of DRb/LSC next to *rps*4. Thus, we infer that the DR in *S. vardei* was caused by this ca. 50-kb inversion, which spans one copy of the DR (*trn*N*-trn*C). In addition, *S. vardei* has an expanded DR with genes originally located in LSC (*psb*M to *trn*C) being included, and *rpo*B was located at the start position of LSC region. Gene *ycf*2 relocated to LSC, and *chl*L/*chl*N relocated to be adjacent to *ycf*1 in *S. vardei*, which was consistent with the plastome of *H. serrata*.

Plastomes of *S. vardei*, *S. indica*, *S. kraussiana*, and *S. tamariscina* have DR structure and are basically syntenic in gene organization (Additional file 3: Figure S3c), whereas those of *S. lepidophylla*, *S. moellendorffii*, and *S. uncinata* possess canonical IR structure, but with rearrangement events among them (Fig. 2 and Additional file 3: Figure S3d, e, f). One remarkable difference between the plastomes of *S. uncinata* and *S. moellendorffii* is that *S. moellendorffii* lacks a ca. 20-kb inversion (from *trn*C to *psb*I), which exists in *S. uncinata* (Fig. 2 and Additional file 3: Figure S3f). This inversion is absent from all other published lycophyte plastomes, suggesting that the absence of this *trn*C-*psb*I inversion might be the ancestral state in lycophytes (Fig. 2). Two inversions (*ndh*J-*psb*D region and *clp*P-*psb*J region) exist in *S. moellendorffii* plastome in comparison with *S. tamariscina* (Additional file 3: Figure S3e). A 65-kb inversion (*trn*D-*psa*C region) exists in *S. lepidophylla* in comparison with *S. vardei* (Additional file 3: Figure S3d).

### Short Dispersed Repeats (SDRs) in Plastomes of *S. vardei* and Other Species

**Fig. 3:**
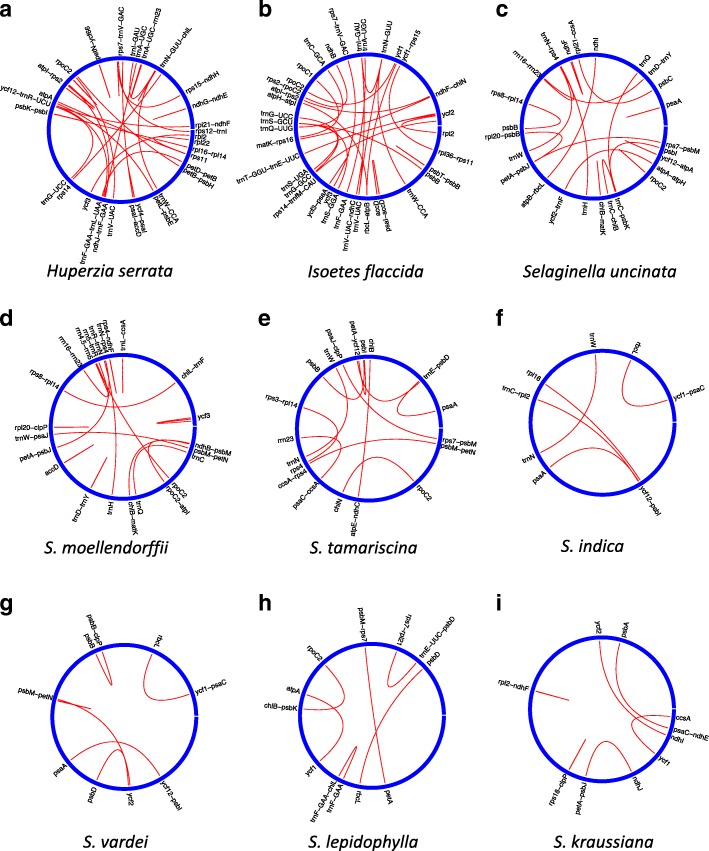
Distribution of Repeats in *S. vardei* and other species in Lycophytes. The gap of each circle represents the start position and the direction is clockwise, panel **a**-**i** represent the layouts of repeats in each species

Repeat analyses showed that fewer SDRs exist in the Selaginellaceae plastomes in comparison with those in other lycophytes (Isoetaceae and Lycopodiaceae; Fig. 3, Table 2 and Additional file 10: Table S5), and many fewer SDRs exist in Selaginellaceae with DR plastid structure in comparison with those with the IR structure. *Huperzia serrata* had the most SDRs (31; Fig. 3a), whereas *S. indica* contained the fewest (5; Fig. 3f). Furthermore, no SDRs were found at the endpoints of the inversions in plastomes with the DR structure, and all the SDRs was basically less than 50 bp, except one in *S. kraussiana*. In the plastome of *S. lepidophylla*, 16 copies of a 17 bp repeat unit dispersed in the intergenic region of *trn*F-*chl*L (Additional file 10: Table S5-9). The 16 copies of repeat units can pair into a number of repeats, which are not displayed in the circular layouts (Fig. 3). In *S. uncinata*, a pair of SDR (46 bp) was located between the *psb*I and the *trn*C-*psb*K intergenic region, flanking the ca. 20-kb inversion from *psb*I-*trn*C (Fig. 3c and Additional file 10: Table S5-3). Another pair of 98 bp SDR located between *pet*A-*psb*J/*rpl*20-*psb*B in plastome of *S. uncinata* could also potentially cause inversions (Fig. 3c and Additional file 10: Tables S5-3).

**Table 2:** Representatives related for confirmation of plastome structure of *S. vardei*

Species	No. SDRs	>=16 bp	>=30 bp	>=50 bp
*S. uncinata*	14	11	1	2
*S. moellendorffii*	18	14	3	1
*S. tamariscina*	11	11	0	0
*S. indica*	5	5	0	0
*S. vardei*	6	6	0	0
*S. lepidophylla*	6	6	0	0
*S. kraussiana*	6	4	1	1
*I. flaccida*	29	22	5	1
*H. serrata*	31	24	6	1

Note: species are listed following phylogenetic relationships

### The Evolution of DR in Plastomes of Land Plants

**Fig. 4:**
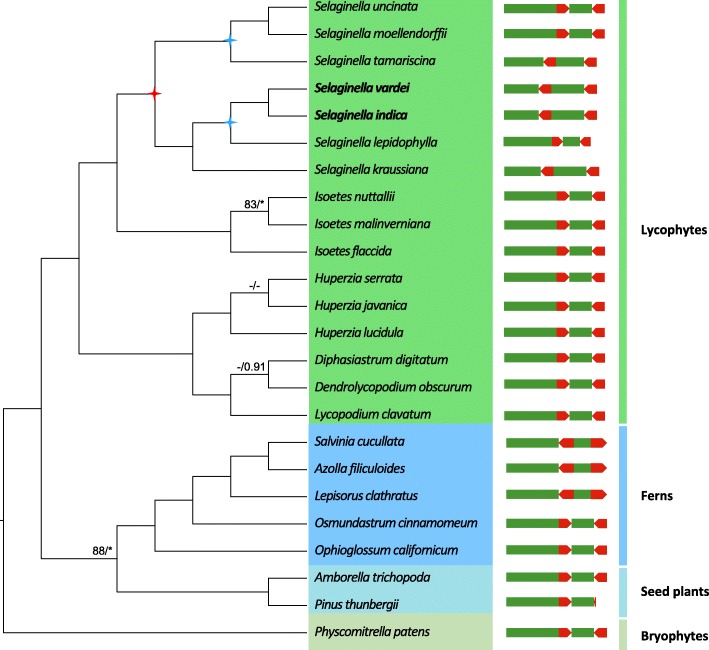
Phylogenetic reconstruction and simplified structure of plastomes of each species. The red star represents the origination of DR structure, whereas the blue stars represent the independent origination of IR structure in Selaginellaceae

Phylogenetic relationships based on 32 plastid protein-coding genes (Fig. 4) showed that subg. *Rupestrae* containing *S. vardei* and *S. indica*, subg. *Lepidophyllae* and subg. *Gymnogynum* formed into one clade and was sister to the remaining *Selaginella* species with sequenced plastomes (*S. tamariscina*, *S. moellendorffii* and *S. uncinata*), which is congruent with the recent published phylogenies of Selaginellaceae [29, 30] (Fig. 4). When we mapped the simplified structure of plastomes onto the phylogenetic tree, it is clear that most sequenced land plant plastomes possess the typical IR structure. Compared to the plastomes of the early diverged ferns, such as Hymenophyllales, Marattiales, Psilotales, Ophioglossales, and Equisetales, the rRNA genes in the IR region were arranged in reverse order in Schizaeales and core leptosporangiates [31–33], with plastomes still maintaining the IR structure. To sum up, the plastomes with a DR structure only occurred in Selaginellaceae (Fig. 4).

## Discussion

### The Characterization of the DR Structure in the Plastomes of Selaginellaceae

The plastomes with DR structure, first reported in a red alga [22], originated independently in the phylogenetically distant Selaginellaceae. Considering the ancestral plastome in lycophytes (Lycopodiopsida; PPG 2016 [34]) likely has the canonical IR as shown in plastomes of Lycopodiaceae [35–37] and Isoetaceae [38], we propose that the innovative DR structure of plastomes in Selaginellaceae [23, 24] originated in the stem lineage of the family.

The DR structure in *S. vardei*, *S. indica*, and *S. dregei* (Table 1, Additional file 2: Figure S2), suggest that the DR structure is a shared characteristic in subg. *Rupestrae*. Following the infrageneric classification of Weststrand and Korall [25], plastomes from subg. *Gymnogynum* (*S. kraussiana*) and subg. *Rupestrae* (*S. vardei* and *S. indica*) possess the DR structure, subg. *Lepidophyllae* (*S. lepidophylla*) possesses the IR structure, whereas subg. *Stachygynandrum* possesses both DR (*S. tamariscina*) and IR (*S. moellendorffii* and *S. uncinata*) structure. Therefore, two primary potential explanations exist for DR structure origin: 1) DR structure is the ancestral state of Selaginellaceae plastomes with at least two independent origins of IR structure (in *S. lepidophylla* and the clade including *S. moellendorffii* and *S. uncinata*); 2) IR structure is the ancestral state and at least three independent origin events are necessary for the three clades possessing DR structure (or two evolutions of DR and a subsequent re-evolution of IR). Since the gene organization in plastomes with the DR structure is basically congruent, and the organization of the plastomes with the IR structure is divergent (Fig. 2), we propose that the first hypothesis is more reasonable (Fig. 4).

The inversion of ca. 50-kb *trn*N*-trn*F region detected in *S. vardei* and other plastomes with a DR structure presumably resulted in the change to a DR structure. The ca. 50-kb inversion starts from the LSC and terminates at the border of DRb/SSC, spanning one DR copy, thus, results in the orientation change from inverted to direct (Fig. 1).

### Conformation of the Plastome with DR Structure of Selaginellaceae

The standard depiction of the plastome is a genome-sized circular DNA molecule, and plastomes with IR structure are able to generate two potentially equimolar isomers [9, 39]. The two isomers differ only in the relative orientation of their single-copy regions, which was hypothesized to occur through interconversion within the two IR regions intramolecularly [9, 39]. However, it is now recognized that most plastomes display a great structural diversity existing as linear/concatemeric/highly branched complex molecules [40]. The orientation change of single-copy regions has been reinterpreted as the results of a BIR (break-induced replication)-like, recombination-dependent replication (RDR) mechanism among linear plastome templates [17, 41]. However, the recombinational activities of plastomes with DR structure have not been discussed. As proposed in mitogenomes, the existence of direct repeats could promote multipartite chromosomal architecture of master chromosomes and subgenomes [42]. Repeats larger than 1 kb appear to mediate high frequency, reciprocal recombination that can result in subgenomes of the genome in approximately equal stoichiometry (the quantities of DNA molecules in different form) [17], as shown in the mitogenome of *Ginkgo biloba* L. [43]. Thus, progress in understanding the structure of both mitogenomes and plastomes suggest that both linear and circular forms of *Selaginella* plastomes exist in vivo, and recombination between the two copies of DR region could promote the generation of subgenomes following the RDR mechanism, with master chromosomes and subgenomes potentially occurring at similar stoichiometries. Two alternative read assemblies mapped at both ends of two copies of DR region reflect the existence of subgenomes in the plastome of *S. vardei* (Additional file 4: Figure S4). Furthermore, we screened the PacBio reads of the *S. tamariscina* plastome from GenBank and selected seven reads spanning the whole DR with both ends adjacent to genes from the LSC, and two reads spanning the DR with both ends adjacent to genes of the SSC (Additional file 5: Figure S5) confirming the recombination between DR regions and the existence of subgenomes in plastomes with DR structure. Either the master chromosome or subgenomic chromosomes could form head-to-tail multimers or branched complexes based on the RDR mechanism [40] (Additional file 4: Figure S4**e**).

### The Co-occurrence of DR structure with reductions in SDRs in the Plastomes of Selaginellaceae

Remarkably, there are extremely few SDRs in Selaginellaceae plastomes with DR structure (Fig. 3). No SDRs are located at the ends of this ca. 50-kb inversion and the size of almost all the SDRs is less than 30 bp in plastomes with DR structure. Repeats less than 50 bp is only possibly able to invoke microhomology-mediated recombination, whereas as repeats larger than 50 bp is mostly able to mediate homologous recombination intermolecularly and intermolecularly in plastomes and mitogenomes [17]. A low frequency of illegitimate recombination can be presumably induced by the micro-homologous repeats (less than 30 bp) only at the absence of plant-specific single-strand DNA (ssDNA)-binding protein the Whirlies, which is related to the recombination surveillance machinery [44]. In addition to the recombination of DR region, other repeats large enough to recombine efficiently often result in unintended secondary homologous recombination events [45]. The secondary recombination caused by direct or inverted short repeats could result in plastome fragmentation or gene loss, which may have the potential to destabilize the plastome of land plants [17]. Therefore, we hypothesize that plastomes with DR structure are possibly susceptive to SDRs scattered in single copy regions [43, 46], thus, the recombinational SDRs are selected against to maintain the integrity and stability of plastomes.

## Conclusions

We documented the unconventional DR structure in plastomes of subg. *Rupestrae* and hypothesize that the DR structure is the ancestral state of Selaginellaceae plastomes. We propose that the ca. 50-kb *trn*N-*trn*F inversion resulted in the DR structure, and the DR regions could mediate the recombination activity, generating approximately equimolar subgenomes. Subsequently, the extreme reduction in SDRs of lineages with the DR structure is presumably the result of selection to avoid the potential secondary recombination and plays a key role in maintaining the integrity and stability of the unconventional plastomes with DR structure.

## Materials and Methods

### Taxon Sampling, DNA Extraction, Sequencing and Assembly

*Selaginella vardei* is a member of the monophyletic subg. *Rupestrae sensu* Weststrand and Korall [25], characterized by having monomorphic and helically arranged vegetative leaves and tetrastichous strobili. We collected a sample of *S. vardei* from the wild in Sichuan Province and the closely related *S. indica* from Yunnan Province for this study and deposited the voucher specimens of this collection in the Herbarium of Institute of Botany, CAS (PE) (Table 1).

Total genomic DNA was isolated from silica gel-dried materials with a modified cetyl- trimethylammonium bromide (CTAB) method [47]. Library construction was performed with the NEBNext DNA Library Prep Kit (New England Biolabs, Ipswich, Massachusetts, USA) and sequencing was performed on the Illumina HiSeq 2500 (Illumina, San Diego, California, USA). Illumina paired-end reads of 150 bp were mapped to *S. uncinata* (AB197035) [27] and *S. moellendorffii* (FJ755183) [28], with medium-low sensitivity in five to ten iterations in Geneious 9.1.4 (Biomatters, Inc., Auckland, New Zealand; https://www.geneious.com) [48]. The mapped reads were then assembled into contigs in Geneious. Additionally, the cleaned reads were assembled de novo with SPAdes v. 3.10.1 [49] using a range of kmer sizes from 21 to 99. Putative plastome contigs were identified using BLASTN 2.2.29 [50], with the previously published *S. uncinata* and *S. moellendorffii* plastomes as reference. We also used bandage v. 0.8.1 [26], a program for visualizing de novo assembly graphs, to help select plastome contigs and analyze de novo assembly results by importing the fastg file created by SPAdes. The contigs obtained above were then combined and imported into Geneious to extend and assemble into the complete plastomes.

### Gene Annotation

Gene annotation was performed using local BLAST with default parameter settings [51]. Putative start and stop codons were defined based on similarity with genes of published plastomes [27, 28]. The tRNAs were verified using tRNAscan-SE version 1.21 [52] and ARAGORN [53]. Circular and linear genome maps were drawn with OGDraw version 1.2 [54].

### PCR Confirmation of *S. vardei* Plastome

To confirm the accuracy of our *S. vardei* plastome assembly, we designed 12 primers pairs (Additional file 7: Table S2) using Primer v 3.0 [55] based on the assembled sequence of *S. vardei* for long-range PCR. We also designed five additional primers pairs (Additional file 9: Table S4) at the boundaries of the DR structure and the ca. 50-kb inversion (marked on Fig. 1) to confirm the accuracy of assembly. Both positive (50-kb inversion and DR structure) and negative (without 50-kb inversion and IR structure) controls were carried out using these five primer pairs (Additional file 1: Figure S1b). The PCR amplifications were performed in a total volume of 20 μL containing 4 μL of 5X PrimeSTAR GXL Buffer, 1.6 μL of dNTP Mixture (2.5 mM each), 1.2μL of each primer (5 mM), 0.4 μL of PrimeSTAR GXL DNA Polymerase and 20 ng of template DNA. Cycling conditions were 98 °C for 3 min, followed by 40 cycles of 98 °C for 10 s, 58 °C for 30 s and 72 °C for 5 min for long-range PCR and 1.5 min for normal PCR, and a final extension of 72 °C for 10 min. The PCR products were verified by electrophoresis in 0.8% agarose gels stained with ethidium bromide. Then, we designed internal primers (Additional file 8: Table S3) to get sequences of these long-range PCR products using Sanger sequencing. The PCR products of the DR and inversion confirmation were sequenced by Majorbio, Beijing, China.

### PCR Confirmation of DR Structure in Related Representatives of subg. *Rupestrae*

To further test whether the structure found in *S. vardei* existed in other species of subg. *Rupestrae*, PCR amplification using primers designed at the boundaries of the DR structure and the ca. 50-kb inversion in plastome of *S. vardei* (marked on Fig. 1, Additional file 9: Table S4) were carried out with another individual of *S. vardei*, two individuals of *S. indica*, and one individual of *S. dregei* (Table 1). Only positive control was carried out in these species. The PCR procedure follows the conditions of normal PCR mentioned above. The PCR products were verified by electrophoresis in 0.8% agarose gels stained with ethidium bromide. The PCR products were sequenced by Majorbio, Beijing, China.

### Comparison of the Plastomes of *S. vardei* and Other Lycophytes

Dot plot analyses of the plastomes of *S. vardei* and other lycophytes (*S. lepidophylla*, *S. kraussiana*, *S. tamariscina*, *S. uncinata*, *S. moellendorffii*, *Isoetes flaccida*, and *Huperzia serrata*) were performed using Gepard [56] in order to identify the putative structural rearrangements in the *S. vardei* plastome. The syntenic analyses of linear plastome maps of *S. vardei* and other lycophytes were carried out based on the dot plot analyses. The first site of the LSC at the border of the LSC/IRa was considered as the starting point.

### Repeat Analyses

Short dispersed repeats (SDRs) were identified using RepeatsFinder [57] with default parameters. The circular layout of SDRs in plastomes was then visualized using the *circlize* package [58] in R v. 3.4.1 (R Development Core Team2012). The locations of SDRs were marked outside the circle in order to find possible correlations with rearrangements. Nine plastomes (*S. lepidophylla*, *S. vardei*, *S. indica*, *S. kraussiana*, *S. tamariscina*, *S. uncinata*, *S. moellendorffii*, *I. flaccida* and *H. serrata*) were included. One copy of the IR/DR was removed from all plastomes used.

### Phylogenetic Analyses

Thirty-two conserved plastid protein-coding genes were used to reconstruct the phylogenetic framework using 19 species from previously published plastomes of land plants (from bryophytes to seed plants; Additional file 11: Table S6). For *S. tamariscina*, we downloaded the raw reads from GenBank (SRR6228814, SRR7135413) [23], assembled plastid contigs and extracted the 32 gene sequences since the complete plastome has not been released on GenBank. A total of 19,248 bp sequences were aligned at the protein level by MAFFT [59] using the translation-aligned function in Geneious v. 9.1.4. Poorly aligned regions were removed using Gblocks v. 0.91b [60]. Maximum-likelihood (ML) analysis was performed using RAxML v. 7.4.2 with 1000 bootstrap replicates and the GTR+G model [61] based on Akaike information criterion (AIC) in jModeltest 2.1.7 [62]. The simplified structure of the plastome of each species (Additional file 11: Table S6) was then mapped on the phylogenetic tree showing the direction of rRNA-encoding repeat.

## Additional files

Additional file 1: **Figure S1.** Confirmation of *S. vardei* plastome structure. **a:** Long-range PCR amplification results of *S. vardei*. Primer pairs used are indicated at the top of each lane. Size markers are in bp. Gene names of these 12 products are as follows: 1: *rpo*B – *rps*2; 2: *rps*2 – *chl*B; 3: *chl*B – *ycf*2; 4: *ycf*2 – *psa*B; 5: *psa*B – *rrn*23; 6: *rrn*23 – *rpl*2; 7: *rpl*2 – *pet*B; 8: *pet*B – *pet*E; 9: *pet*E – *atp*B; 10: *atp*B – *ycf*1; 11: *ycf*1 – *rrn*23; 12: *rrn*23 – *rpo*B. **b:** PCR confirmation results of DR structure and Inversion. Primer pairs used are indicated at the top of each lane. Size markers are in bp. Gene names of these 12 fragments are as follows: positive control: 1-2, *rps*4 – *rrn*5; 3-4, *pet*N – *rpl*2; 1-2, *rps*4 – *rrn*5; 5-6, *atp*E – *chl*L; 7-8, *ccs*A – *rrn*5; 9-10, *pet*N – *rpo*B; negative control: 1-3, *rps*4 – *pet*N; 2-4, *rrn*5 – *rpl*2; 1-5, *rps*4 – *atp*E; 2-6, *rrn*5 – *chl*L; 7-9, *ccs*A – *pet*N; 8-10, *rrn*5 – *rpo*B; **c:** Reads coverage of *S. vardei* plastomes; **d:** The assembled graph in Bandage showing DR structure of *S. vardei*. (PDF 175 kb)

Additional file 2: **Figure S2.** Plastome structure confirmation and sequence alignments of related representatives. **a:** PCR confirmation results of DR structure in another individual of *S. vardei* and two representative species within subg. *Rupestrae*. Primer pairs used are indicated at the top of each lane. Size markers are in bp. Species name and gene names of these fragments are as follows: 836: *S. vardei*; 5868: *S. indica*; 6255: *S. indica*; 26698: *S. dregei*; 1-2: *rps*4 – *rrn*5; 3-4: *pet*N – *rpl*2; 5-6: *atp*E – *chl*L; 7-8: *ccs*A – *rrn*5; 9-10: *pet*N – *rpo*B; **b:** Alignment results of sequences from each product in **a**, the light grey color represents regions with identical base pairs among individuals, whereas the dark color highlights regions with mismatched base pairs. (PDF 115 kb)

Additional file 3: **Figure S3.** Dot-plot analyses of plastomes between *S. vardei* and other lycophyte species. (PDF 489 kb)

Additional file 4: **Figure S4.** Reads coverages of putative subgenomes and alternative reads assemblies in *S. vardei*. **a, b:** Two alternative reads assemblies at LSC/DRa and SSC/DRb boundaries: the unmatched reads in black box in **a** is consistent with assembled sequence in black box in **b**, the unmatched reads in red box in **b** is consistent with assembled sequence in red box in **a**; **c, d:** Two alternative reads assemblies at DRa/SSC and DRb/LSC boundaries: the unmatched reads in black box in **c** is consistent with assembled sequence in black box in **d**, the unmatched reads in red box in **d** is consistent with assembled sequence in red box in **c. e:** the simplified structure for master chromosomes and subgenomes based on **a**, **b**, **c**, **d** in this figure. We define the arrow end as B, and the other end as A. End B of either LSC (**a**) or SSC (**b**) can be assembled with end B of DR. End A of either LSC (**c**) or SSC (**d**) can be assembled with end A of DR. (PDF 1549 kb)

Additional file 5: **Figure S5.** Screened PacBio reads of *S. tamariscina* showing evidence of the existence of subgenomes in plastomes with DR. **a:** the simplified plastome structure of *S. tamariscina* based on Xu et al. (2018); **b, c:** the simplified subgenome structure of plastomes with DR, supported by the screened PacBio reads of *S. tamariscina* plastome as listed. (PDF 29 kb)

Additional file 6: **Table S1.** Genes present in the plastome of *S. vardei*. (DOCX 15 kb)

Additional file 7: **Table S2.** Primers designed for long range PCR amplification of plastome of *S. vardei*. (DOCX 14 kb)

Additional file 8: **Table S3.** Internal primers designed for Sanger sequencing of long-range PCR products of *S. vardei*. (DOCX 18 kb)

Additional file 9: **Table S4.** Primers designed for Sanger sequencing of PCR confirmation of *Selaginella* subg. *Rupestrae*. (DOCX 14 kb)

Additional file 10: **Table S5.** Detailed dispersed short repeats in plastomes of each species. (DOCX 45 kb)

Additional file 11: **Table S6.** Species selected in the phylogenetic analyses. (DOCX 15 kb)

## Data Availability

The newly sequenced plastome sequences have been deposited in GenBank under the accession number MG272482 and MK156801.

## Abbreviations

AIC: Akaike information criterion
BIR: Break-induced replication
CTAB: Cetyl-trimethylammonium bromide
DR: Direct repeat
IR: Inverted repeat
LSC: Large single copy
ML: Maximum likelihood
NGS: Next-generation sequencing
RDR: Recombination-dependent replication
rRNA: Ribosomal RNA
SDRs: Short dispersed repeats
SSC: Small single copy
ssDNA: single-strand DNA
tRNA: Transfer RNA
