# Correction to: Directed Repeats Co-occur with Few Short-Dispersed Repeats in Plastid Genome of a Spikemoss, *Selaginella vardei* (Selaginellaceae, Lycopodiopsida)

**DOI:** 10.1186/s12864-019-5919-3

**Published:** 2019-06-26

**Authors:** Hong-Rui Zhang, Xian-Chun Zhang, Qiao-Ping Xiang

**Affiliations:** 1 State Key Laboratory of Systematic and Evolutionary Botany, Institute of Botany, the Chinese Academy of Sciences, Beijing, 100093 China; 2 University of Chinese Academy of Sciences, Beijing, 100049 China

**Correction to: BMC Genomics (2019) 20:484**

**https://doi.org/10.1186/s12864-019-5843-6**

Following the publication of this article [1], the authors reported that the Fig. 2 described in the article had a mistake that two grey blocks in *S. moellendorffii* was not placed as background color, and in the Fig. 2 legend, *chl*L-*chl*N was wrongly written into *chl*L-*chl*L. They have therefore provided the following alternative Fig. 2 in this Correction article in order to show the accurate information.

**Fig. 2:**
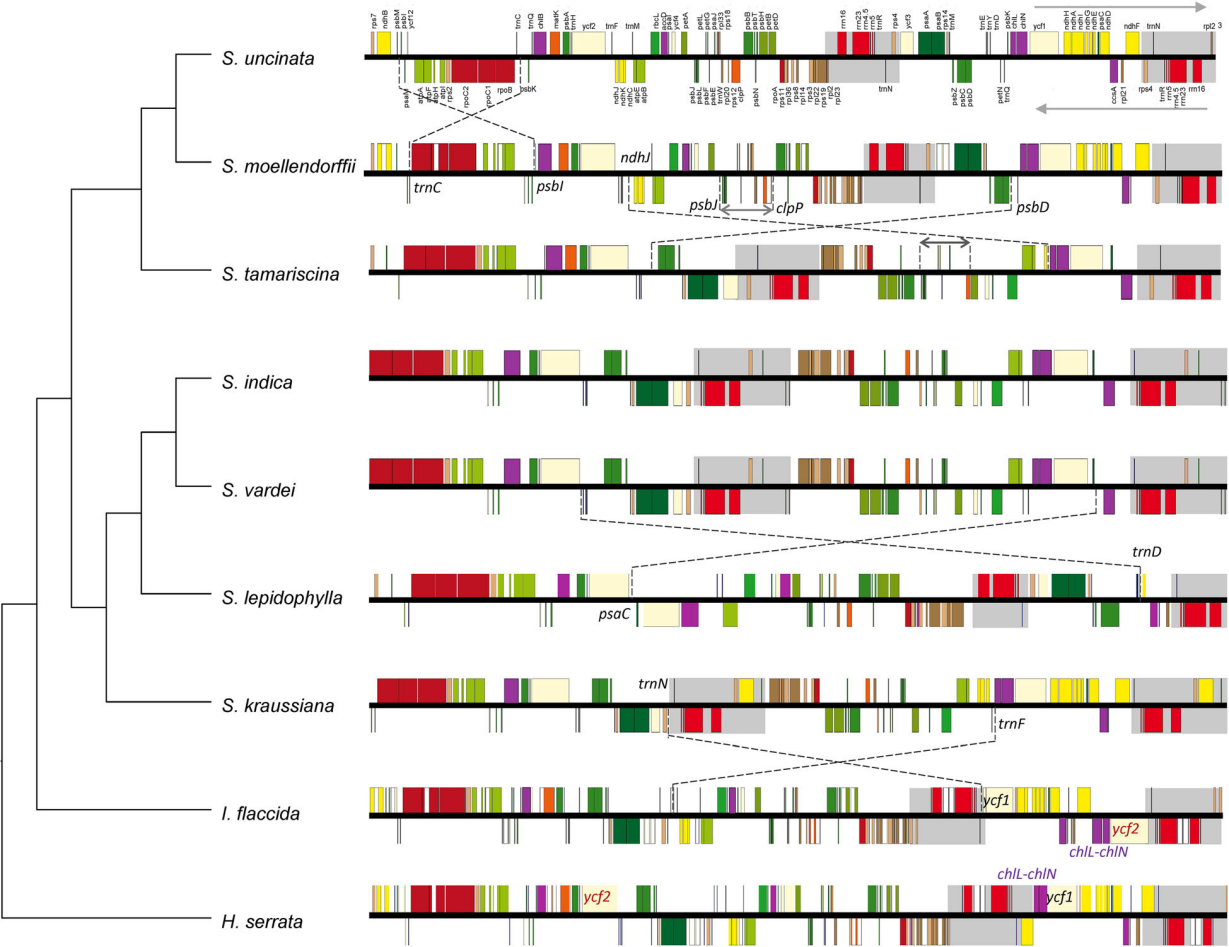
Linear maps of plastomes between *S. vardei* and other lycophytes showing the rearrangements. Upper arrow indicates genes transcribed in forward direction; lower arrow indicates genes transcribed in reverse direction. The red *ycf*2 indicates the translocation from LSC to SSC and the purple *chl*L-*chl*N indicates the inversion in *I. flaccida*

## References

[1] Zhang H-R, Zhang X-C, Xiang Q-P (2019). Directed repeats co-occur with few short-dispersed repeats in plastid genome of a Spikemoss, *Selaginella vardei* (Selaginellaceae, Lycopodiopsida). BMC Genomics.

