# Supplementary figures and images for: Directed Repeats Co-occur with Few Short-Dispersed Repeats in Plastid Genome of a Spikemoss, Selaginella vardei (Selaginellaceae, Lycopodiopsida)
Source: BMC Genomics. 2019 Jun 11;20:484. doi: 10.1186/s12864-019-5843-6 (PMC6560725; doi:10.1186/s12864-019-5843-6)

**a**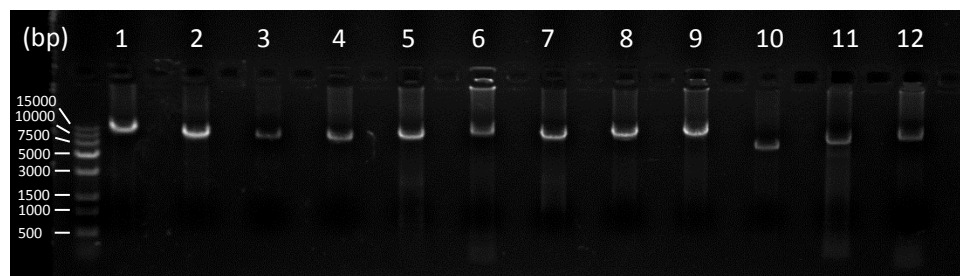**b**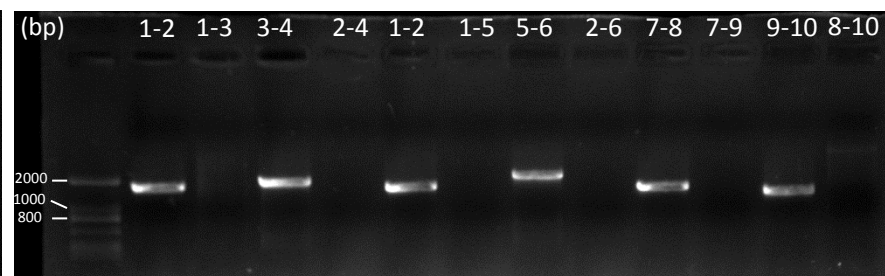**c**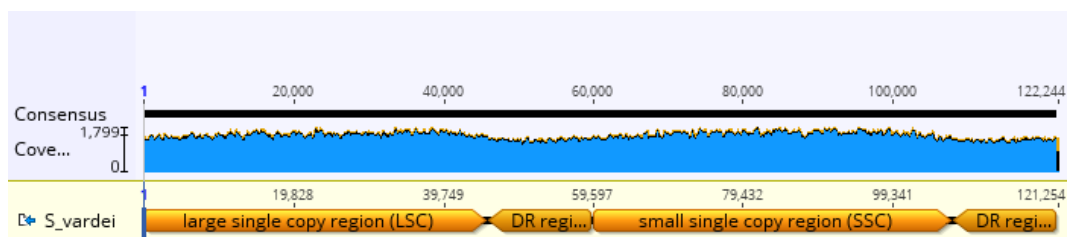**d**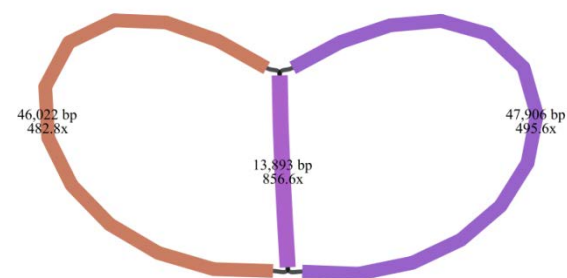

Supplement: Supplementary file 1 — Figure S1. Confirmation of S. vardei plastome structure. a: Long-range PCR amplification results of S. vardei. Primer pairs used are indicated at the top of each lane. Size markers are in bp. Gene names of these 12 products are as follows: 1: rpoB – rps2; 2: rps2 – chlB; 3: chlB – ycf2; 4: ycf2 – psaB; 5: psaB – rrn23; 6: rrn23 – rpl2; 7: rpl2 – petB; 8: petB – petE; 9: petE – atpB; 10: atpB – ycf1; 11: ycf1 – rrn23; 12: rrn23 – rpoB. b: PCR confirmation results of DR structure and Inversion. Primer pairs used are indicated at the top of each lane. Size markers are in bp. Gene names of these 12 fragments are as follows: positive control: 1-2, rps4 – rrn5; 3-4, petN – rpl2; 1-2, rps4 – rrn5; 5-6, atpE – chlL; 7-8, ccsA – rrn5; 9-10, petN – rpoB; negative control: 1-3, rps4 – petN; 2-4, rrn5 – rpl2; 1-5, rps4 – atpE; 2-6, rrn5 – chlL; 7-9, ccsA – petN; 8-10, rrn5 – rpoB; c: Reads coverage of S. vardei plastomes; d: The assembled graph in Bandage showing DR structure of S. vardei. (PDF 175 kb) [file 12864_2019_5843_MOESM1_ESM.pdf]

**a**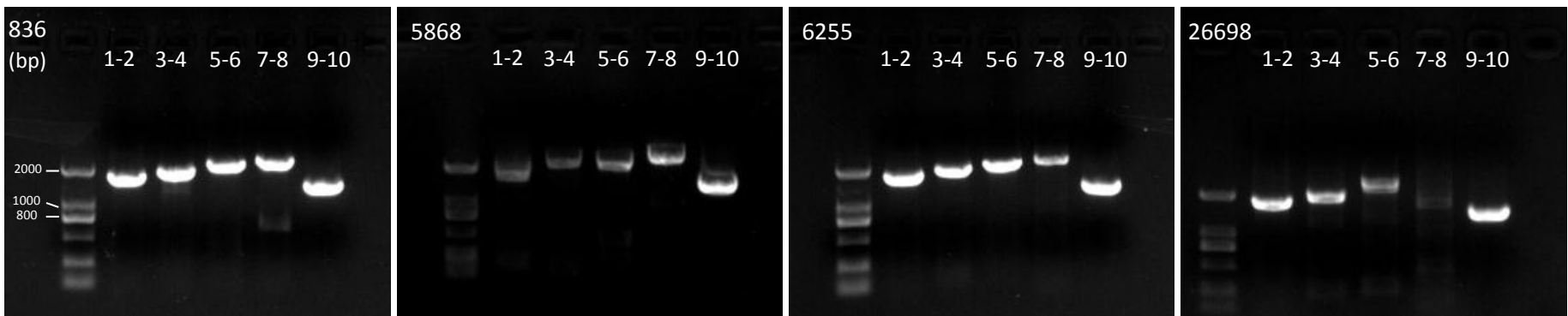**b**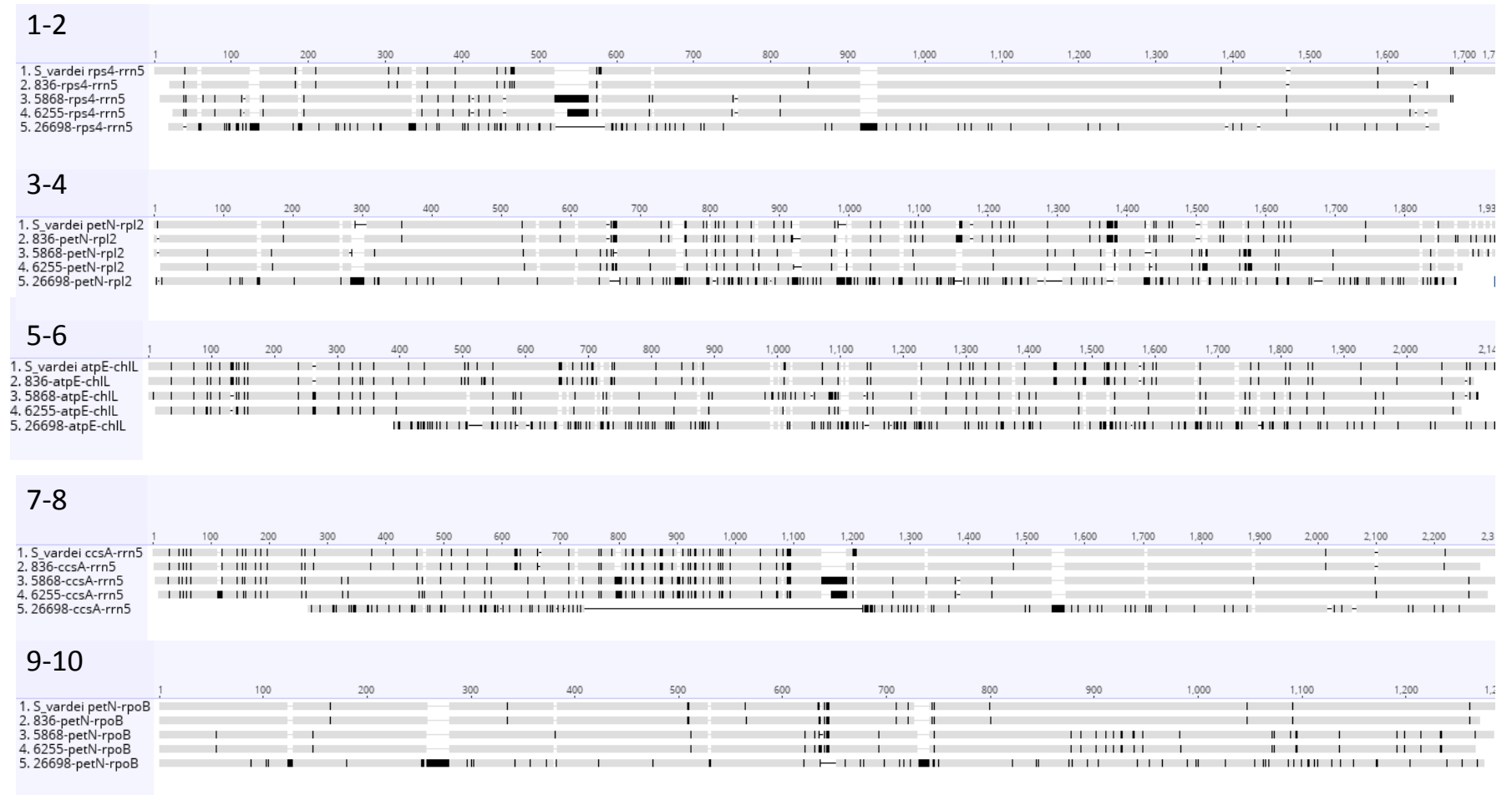

Supplement: Supplementary file 2 — Figure S2. Plastome structure confirmation and sequence alignments of related representatives. a: PCR confirmation results of DR structure in another individual of S. vardei and two representative species within subg. Rupestrae. Primer pairs used are indicated at the top of each lane. Size markers are in bp. Species name and gene names of these fragments are as follows: 836: S. vardei; 5868: S. indica; 6255: S. indica; 26698: S. dregei; 1-2: rps4 – rrn5; 3-4: petN – rpl2; 5-6: atpE – chlL; 7-8: ccsA – rrn5; 9-10: petN – rpoB; b: Alignment results of sequences from each product in a, the light grey color represents regions with identical base pairs among individuals, whereas the dark color highlights regions with mismatched base pairs. (PDF 115 kb) [file 12864_2019_5843_MOESM2_ESM.pdf]

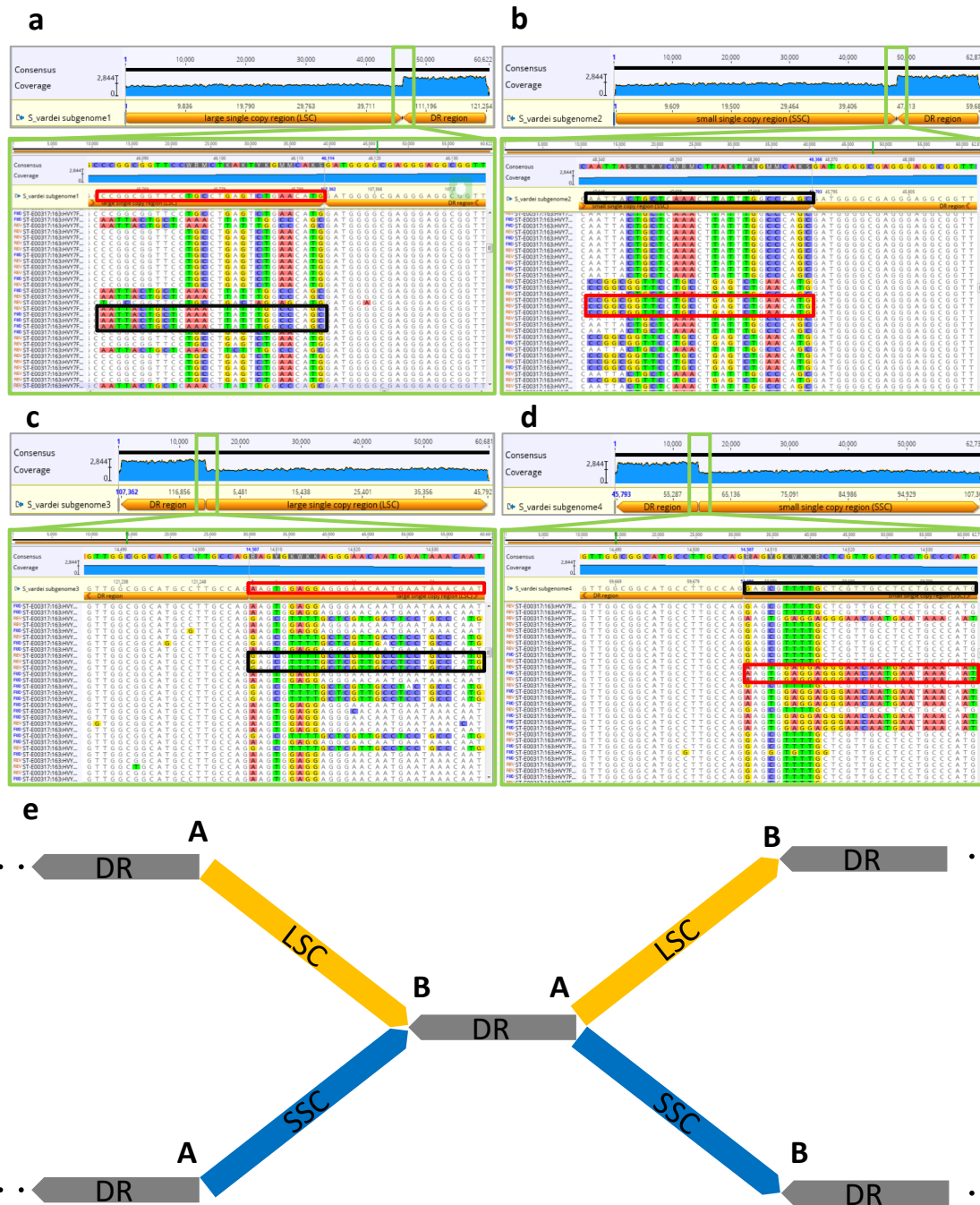

Supplement: Supplementary file 4 — Figure S4. Reads coverages of putative subgenomes and alternative reads assemblies in S. vardei. a, b: Two alternative reads assemblies at LSC/DRa and SSC/DRb boundaries: the unmatched reads in black box in a is consistent with assembled sequence in black box in b, the unmatched reads in red box in b is consistent with assembled sequence in red box in a; c, d: Two alternative reads assemblies at DRa/SSC and DRb/LSC boundaries: the unmatched reads in black box in c is consistent with assembled sequence in black box in d, the unmatched reads in red box in d is consistent with assembled sequence in red box in c. e: the simplified structure for master chromosomes and subgenomes based on a, b, c, d in this figure. We define the arrow end as B, and the other end as A. End B of either LSC (a) or SSC (b) can be assembled with end B of DR. End A of either LSC (c) or SSC (d) can be assembled with end A of DR. (PDF 1549 kb) [file 12864_2019_5843_MOESM4_ESM.pdf]

**a**

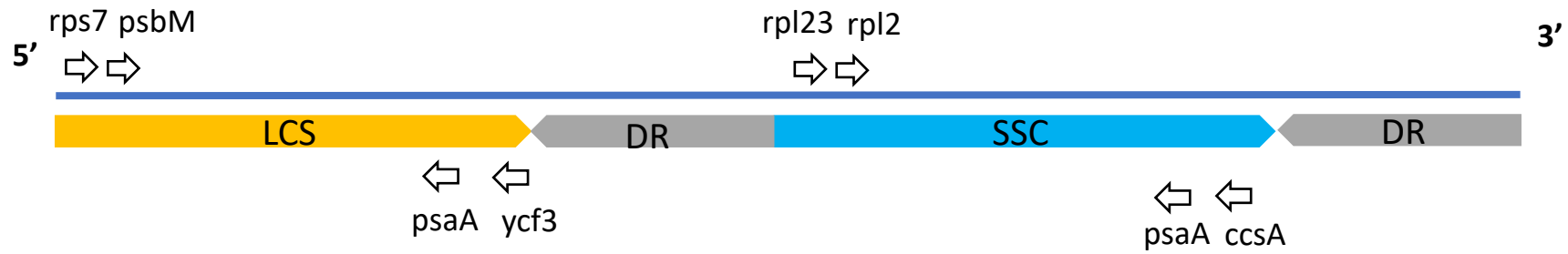

**b**

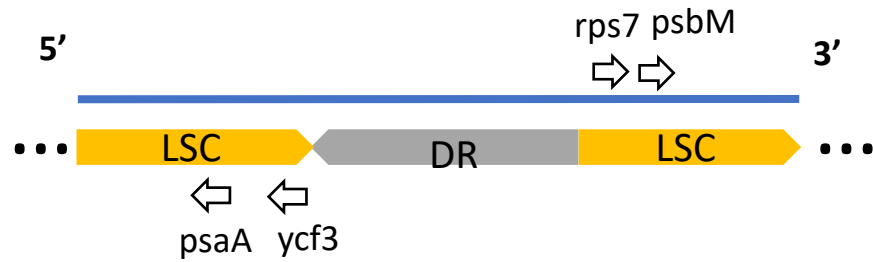

SRR6228814.46005  
SRR6228814.334789  
SRR6228814.584555  
SRR6228814.622792  
SRR6228814.633072  
SRR6228814.1181365  
SRR6228814.1402343

**c**

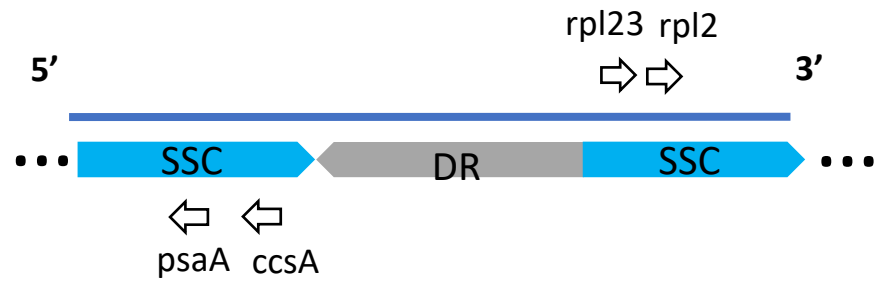

SRR6228814.469138  
SRR6228814.1066297

Supplement: Supplementary file 5 — Figure S5. Screened PacBio reads of S. tamariscina showing evidence of the existence of subgenomes in plastomes with DR. a: the simplified plastome structure of S. tamariscina based on Xu et al. (2018); b, c: the simplified subgenome structure of plastomes with DR, supported by the screened PacBio reads of S. tamariscina plastome as listed. (PDF 29 kb) [file 12864_2019_5843_MOESM5_ESM.pdf]
