# Supplementary material for: Directed Repeats Co-occur with Few Short-Dispersed Repeats in Plastid Genome of a Spikemoss, Selaginella vardei (Selaginellaceae, Lycopodiopsida)
Source: BMC Genomics. 2019 Jun 11;20:484. doi: 10.1186/s12864-019-5843-6 (PMC6560725; doi:10.1186/s12864-019-5843-6)
Supplement: Supplementary file 3 — Figure S3. Dot-plot analyses of plastomes between S. vardei and other lycophyte species. (PDF 489 kb) [file 12864_2019_5843_MOESM3_ESM.pdf]

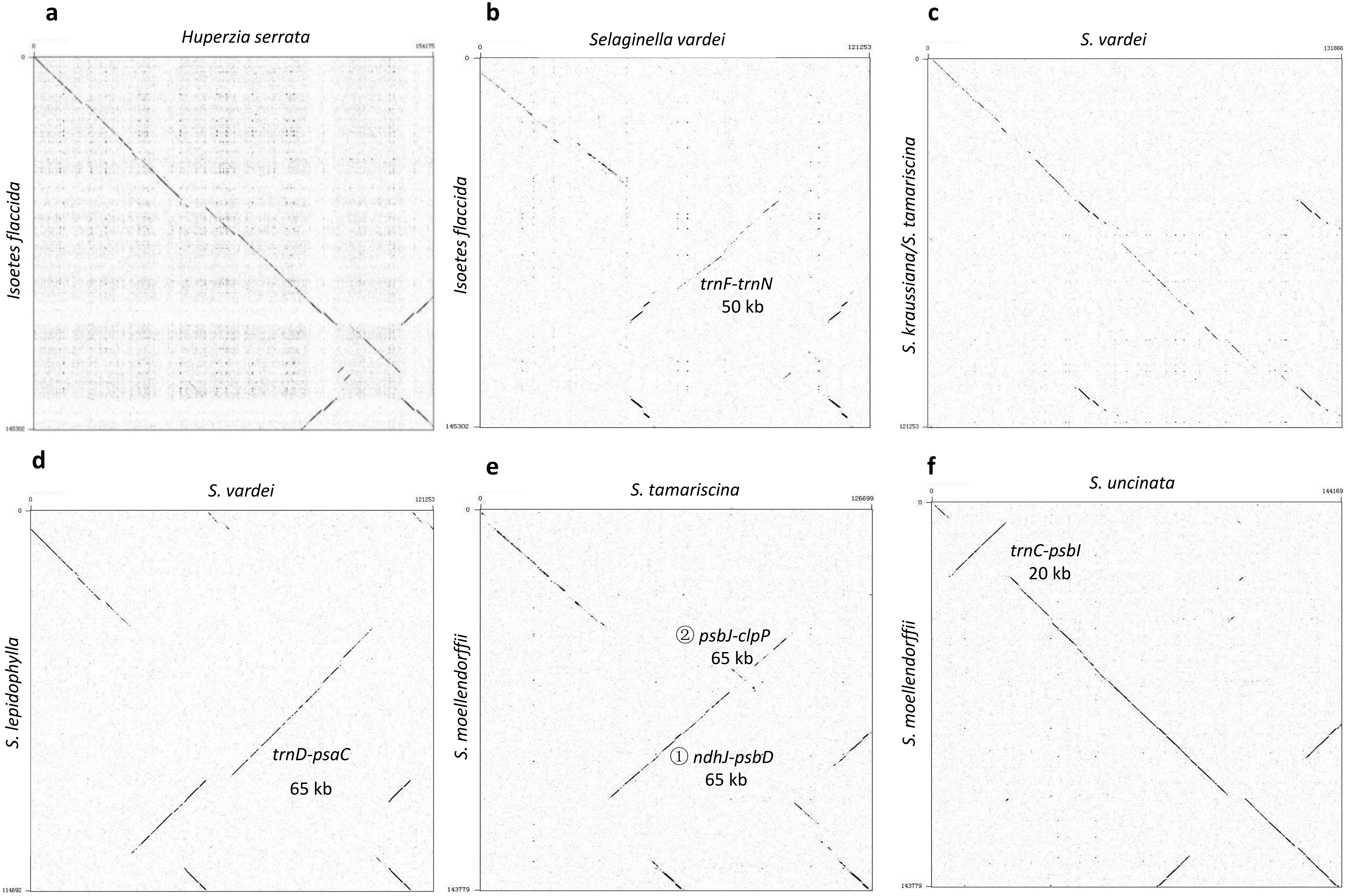
a
Huperzia serrata
154175
Isoetes flaccida
145302
b
Selaginella vardei
121253
Isoetes flaccida
145302
trnF-trnN
50 kb
c
S. vardei
131866
S. kraussiana/S. tamariscina
121253
d
S. vardei
121253
S. lepidophylla
114692
trnD-psaC
65 kb
e
S. tamariscina
126699
S. moellendorffii
143779
② psbJ-clpP
65 kb
① ndhJ-psbD
65 kb
f
S. uncinata
144169
S. moellendorffii
143779
trnC-psbI
20 kb
