# Supplementary material for: Directed Repeats Co-occur with Few Short-Dispersed Repeats in Plastid Genome of a Spikemoss, Selaginella vardei (Selaginellaceae, Lycopodiopsida)
Source: BMC Genomics. 2019 Jun 11;20:484. doi: 10.1186/s12864-019-5843-6 (PMC6560725; doi:10.1186/s12864-019-5843-6)
Supplement: Supplementary file 6 — Table S1. Genes present in the plastome of S. vardei. (DOCX 15 kb) [file 12864_2019_5843_MOESM6_ESM.docx]

Table S1 Genes present in the plastome of *S. vardei*.

| **Chloroplast genome feature** | **Genes** |
| --- | --- |
| Photosystem I | *psaA, psaB, psaC, psaI, psaJ* |
| Photosystem II | *psbA, psbB, psbC, psbD, psbE, psbF, psbH, psbI, psbJ, psbK, psbL, psbM*^2^*, psbN, psbT, psbZ* |
| Cytochrome *b_6_ /f* | *petA, petB*^1^*, petD*^1^*, petG, petL, petN*^2^ |
| ATP synthase | *atpA, atpB, atpE, atpF, atpH, atpI* |
| RuBisCO | *rbcL* |
| Large subunit ribosomal proteins | *rpl2, rpl16, rpl20, rpl21, rpl22, rpl36* |
| Small subunit ribosomal proteins | *rps2, rps3, rps4, rps7*^2^*, rps8, rps11, rps14, rps18, rps19* |
| RNA polymerase | *rpoA, rpoB, rpoC1*^1^*, rpoC2* |
| Unknown function protein-coding gene | *ycf1, ycf2, ycf3*^1^*, ycf4, ycf12* |
| Other genes | *ccsA, chlB, chlL, chlN, clpP, infA* |
| Ribosomal RNAs | *rrn16*^2^*, rrn23*^2^*, rrn4.5*^2^*, rrn5*^2^ |
| Transfer RNAs | *trnC-GCA*^2^*, trnD-GUC, trnE-UUC, trnF-GAA, trnfM-CAU, trnH-GUG, trnM-CAU, trnN-GUU*^2^*, trnR-ACG, trnW-CCA, trnY-GUA* |

^1^ Genes containing a single intron.

^2^ Two gene copies in IRs.
