# Supplementary material for: Directed Repeats Co-occur with Few Short-Dispersed Repeats in Plastid Genome of a Spikemoss, Selaginella vardei (Selaginellaceae, Lycopodiopsida)
Source: BMC Genomics. 2019 Jun 11;20:484. doi: 10.1186/s12864-019-5843-6 (PMC6560725; doi:10.1186/s12864-019-5843-6)
Supplement: Supplementary file 7 — Table S2. Primers designed for long range PCR amplification of plastome of S. vardei. (DOCX 14 kb) [file 12864_2019_5843_MOESM7_ESM.docx]

Table S2. Primers designed for long range PCR amplification of plastome of *S. vardei*.

| **Primers** | **Sequences (5'to3')** |
| --- | --- |
| 1-*rpo*B-F | ATTTCCCAAGGATCGAGGATATGG |
| 1-*rps*2-R | CGAGCGGTTTGGGTTAGATCTATA |
| 2-*rps*2-F | CACTACGTGAATGAGAAATGGCTC |
| 2-*chl*B-R | CTGGAATGATTTGATTAACCCCGG |
| 3-*chl*B-F | CGAGGGTACTAATCAAATAGCGGA |
| 3-*ycf2*-R | TTAGCAATTGATTTATTGGGGGCC |
| 4-*ycf2*-F | ATGCCCCTACCTGCCATATTATTT |
| 4-*psa*B-R | CCGGTAGCTCTTTCCATAGTACAA |
| 5-*psa*B-F | ATTCAGCGTTCTTGAACCAGGATA |
| 5-*rrn*23-R | ACACAGGTGGGTAGGTAGAGAATA |
| 6-*rrn*23-F | ATCTCCGGATCCATGCTTATTTGT |
| 6-*rpl*2-R | TCGGGGTGCAAAATATAACCTTTG |
| 7-*rpl*2-F | CCTAATATCCCAAAACTGCCTTGC |
| 7-*pet*B-R | TTACCAGCAAATATGTCCCTCCTC |
| 8-*pet*B-F | GTGTTGACATGAGGAGGGACATAT |
| 8-*psbE*-R | TCATCGACTTGCTCCGACGAATTG |
| 9-*psbE*-F | ATCCATAGCATCACCATACCTTCC |
| 9-*atpB*-R | TTCATTTGACCATAAACCAAGGCC |
| 10-*atpB*-F | AGACCCCTTGGATTCAACTTCTAC |
| 10-*ycf*1-R | ACTAATAATGCCACTCAGGGGTTT |
| 11-*ycf*1-F | TTCGATTGTGGCTATACATAGGGG |
| 11-*rrn*23-R | CTAGGGATAACAGGCTGATCTTCC |
| 12-*rrn*23-F | ATCTCCGGATCCATGCTTATTTGT |
| 12-*rpoB*-R | CCATATCCTCGATCCTTGGGAAAT |
