# Supplementary material for: Directed Repeats Co-occur with Few Short-Dispersed Repeats in Plastid Genome of a Spikemoss, Selaginella vardei (Selaginellaceae, Lycopodiopsida)
Source: BMC Genomics. 2019 Jun 11;20:484. doi: 10.1186/s12864-019-5843-6 (PMC6560725; doi:10.1186/s12864-019-5843-6)
Supplement: Supplementary file 8 — Table S3. Internal primers designed for Sanger sequencing of long-range PCR products of S. vardei. (DOCX 18 kb) [file 12864_2019_5843_MOESM8_ESM.docx]

Table S3 Internal primers designed for Sanger sequencing of PCR products of *S. vardei*.

| Primers | Primer sequences |
| --- | --- |
| 1-1 F | ATACTATGCGCGTAGGAATGAACA |
| 1-2 F | AGGACTCCGTACTTATTAGCGAAC |
| 1-3 F | GAAATGCCCACTAGAAAATCCGAC |
| 1-4 F | AGATCATTCGTCCAGGGTTCATAG |
| 1-5 F | TAGGCGGGGGTAAAATGATTAGTT |
| 1-6 F | CCATGGATGATTTGCGGATAATGA |
| 1-7 F | GAATTGTGGATATTGCTACGCGAA |
| 1-8 F | GTCCCGACCCTTTATATGTTCGTT |
| 1-9 F | TCTCCCCTCGTACAATACTATCCA |
| 1-10 F | AGATACTATTTTCCAGGGCCTTCC |
| 2-1 F | GGGACCTAGCTAGAACTCAGATTG |
| 2-2 F | TATGAATGACTCTACCACTCGCAC |
| 2-3 F | TCTACGGATTAGTCGTAGCTTTGG |
| 2-4 F | GTTCAAGCGACGAATTACAATTGC |
| 2-5 F | TTACGACGATCTTACCAAACAAGC |
| 2-6 R | ATGAGTTCAGAAGTTATTGCGCAG |
| 2-7 R | CATACGGTGGTTATTTCCCTCGTT |
| 2-8 R | ATCACCCGAAGTTTATGCGATTTC |
| 2-9 F | TTGAGTATGCGGTATTCCCTAAGG |
| 3-1 F | TGGATGGTGGTTCTCAATTCACTT |
| 3-2 R | TGATGGCACCGGAGATAATATTGT |
| 3-3 R | AGTGTTTATCATGTGCCTCTGGTA |
| 3-4 F | ACAATATTATCTCCGGTGCCATCA |
| 3-5 F | CATCCGCCGGAAAGAGAGAG |
| 3-6 R | AGCTTGCCCCTATGGTTCAC |
| 3-7 F | TGCTCAATATCCGCCACGAG |
| 3-8 R | AATTGGCGAGTCCGGTCAAT |
| 3-9 R | GAGAAGGAGGAGGTGGAGGT |
| 3-10 R | CATATCCATGTGCGGTTCCATATG |
| 4-1 F | CGAAATTCAACCGGGTCCATTAAA |
| 4-2 F | TGAGCTTTTCCATCCACCGG |
| 4-3 F | CAAACCATGTCCTTGCCACG |
| 4-4 R | CCACAGTAGCGGCAGAGAAT |
| 4-5 F | GTTGCAACTTTCTCACCGCC |
| 4-6 R | AGGAAAGTGGAGGCTTGAGC |
| 4-7 F | AACACAGCTTATCCCAGTGAATCT |
| 4-8 R | GATACCCACCAATAAGACTAGGCC |
| 4-9 R | GAGATGGGAGATTAGCAGGGAATT |
| 4-10 R | CCGGTAGCTCTTTCCATAGTACAA |
| 5-1 F | TAGCCTTATCCGGTATCAAACGAG |
| 5-2 F | CTTAGCCATGCCTCGTAATTTGAG |
| 5-3 R | CGCGCTTAGAAACTGATCCTTATG |
| 5-4 R | TAAGACCTTTACAATCGTAGCGGA |
| 5-5 R | GGCGAGACAACTGGTTACTCATAA |
| 5-6 F | TTTCCTTGCCCTTCTTACGAGTAA |
| 5-7 R | GAACTCGGTGGTGAAACTCTACTG |
| 5-8 F | TTCCTCTACGACTTAGACACCAGA |
| 5-9 R | TGCAGCTGAGGCATCCTAAC |
| 6-1 F | CTATGGGGTATTAGCAGCCGTTTC |
| 6-2 F | ATTCGTTATCCATCCCACGTAGAG |
| 6-3 R | CCTGATAGGTCGATCCGCTCATAC |
| 6-4 R | ATGCGGGGATATTTACTTCTTCCG |
| 6-5 R | GAGTGTTCAAGCTCTGTCTGTAGT |
| 6-6 R | TAGTGACTGGATGGCGGATACATG |
| 6-7 R | TTCTCGTTCATCCCGGGCTAGATA |
| 6-8 R | GGATATAGTCAATACTGCTTGGGC |
| 6-9 F | GGAAATATAAATTGAGGCAGCCCA |
| 6-10 F | ATCCCACGCCTTACCACTTG |
| 7-1 R | GGATAAATCTTTGGTTGTGCGGAC |
| 7-2 R | GCTGCGCAAATAGGAATCTATCTG |
| 7-3 R | CTCTATTTGCCTAGACGTGATCCA |
| 7-4 F | ATACTCTATGAGATGGGCGGAGTA |
| 7-5 F | CCCTTGACAGTATGATAACCGCTA |
| 7-6 F | GATTTGTGAGAATTGCCGGCTAAT |
| 7-7 F | CAGGACATTGTATTGCCACCTTTC |
| 7-8 R | GTCCTAGATCCAGCTATGATTGGG |
| 7-9 F | AGGGTACTGTCAGTGGTCCC |
| 8-1 F | GTGTTGACATGAGGAGGGACATAT |
| 8-2 R | GAATAATCTTCTCCGCTACACCCT |
| 8-3 R | TATCTGAAGCTTGGTCCAGAATCC |
| 8-4 F | TGGACGGGTCATAAAGGGTATAAC |
| 8-5 F | CCTTAGTTTATTGGTAGCGGGGTA |
| 8-6 F | CAAGATTTATGCACAGAGAACGGG |
| 8-7 R | TGCCCAAACAAATGAATGGATTGA |
| 8-8 F | CCTCCGAAGCAGACTCATATTCTT |
| 8-9 R | GTCGTTACATGAGCCGACCTATAT |
| 8-10 F | CTGGAGTTGTCGGGGTCAAA |
| 9-1 F | GAAGGATTCCTCTGTGGCTGATAG |
| 9-2 R | AGGAGGAGCAACAATACAGTGTAC |
| 9-3 F | TACACTGTATTGTTGCTCCTCCTT |
| 9-4 F | AATTGGGGGAGGTATACGATTCAG |
| 9-5 F | TAGTTGTCCCGATCCGAGTTAATC |
| 9-6 F | CCATAAGTAAGAGCCGGAAAACAC |
| 9-7 F | GGATAATATTTTGCCGAACCTCCC |
| 9-8 R | TCACTTTAGGTTTCGTGGATTTGC |
| 9-9 R | CTCCCGACTACGTGACCAAG |
| 9-10 F | AGTCATTCGGAATGCTGCCA |
| 10-1 F | GCGTGATGACCCCTAACCAA |
| 10-2 F | TCCGTCGGCCCATTAATGAG |
| 10-3 F | TTACGAGTCAAGGCCTGTGC |
| 10-4 R | TCCGCGGAAATCATGACTCC |
| 10-5 F | GTCATTCCCCACAAAACGCC |
| 10-6 F | ATTGTTCATGAGGTTGGTATCCCA |
| 11-1 F | TGGGGATACTGGAGTCCCTG |
| 11-2 F | GAGGGGCCACTCACTGATAC |
| 11-3 R | AACCCTGGCGAACTGATTCC |
| 11-4 F | TGTCTACCGGTTTCACCACG |
| 11-5 F | CGGACGGCTGGTAATCATGA |
| 11-6 F | GAAAACTGCAGTTACCCCGC |
| 11-7 F | CCCAGCCCGTCCATTTGTAT |
| 11-8 R | CTAGGGATAACAGGCTGATCTTCC |
| 11-9 F | TGTGCAAAGGGATAGGGATGTTAA |
| 11-10 R | TTAACATCCCTATCCCTTTGCACA |
| 11-11F | ATGTAAAAGGTGTGAATCCGCTTG |
| 12-1 R | TACCCCAGTAGTCCTAGCCG |
| 12-2 F | ATTAGCAGCCGTTTCCAGCT |
| 12-3 R | AACACATGCAAGTCGTACGG |
| 12-4 R | ATGGCGCTACAGGGAATTCC |
| 12-5 F | TGGAATCCGCACGAGGAAAA |
| 12-6 F | ACGGCATCTCTCATCGTTCC |
| 12-7 R | TCGTTCCTTTGCGATTTGGC |
| 12-8 F | GGGTATTATGGGCGATCGCA |
| 12-9 R | TTGTGTTCACAAGCTAGCGA |
| 12-10 R | TCAATACTGCTTGGGCTGCC |
| 12-11 R | TACCACCCTCTGGCAATGTG |
