# Supplementary material for: Directed Repeats Co-occur with Few Short-Dispersed Repeats in Plastid Genome of a Spikemoss, Selaginella vardei (Selaginellaceae, Lycopodiopsida)
Source: BMC Genomics. 2019 Jun 11;20:484. doi: 10.1186/s12864-019-5843-6 (PMC6560725; doi:10.1186/s12864-019-5843-6)
Supplement: Supplementary file 9 — Table S4. Primers designed for Sanger sequencing of PCR confirmation of Selaginella subg. Rupestrae. (DOCX 14 kb) [file 12864_2019_5843_MOESM9_ESM.docx]

Table S4. Primers designed for PCR confirmation of *Selaginella* subg. *Rupestrae*.

| **Primers** | **Sequences (5'to3')** |
| --- | --- |
| 1-*rps4*-F | GGGCATATAACCAGGCTTT |
| 2-*rrn*5-R | GTAGAGGAACCACACCAATC |
| 3-*pet*N-F | GCAGCCCAAGCAGTATTGAC |
| 4-*rpl*2-R | TTGTCACCGTCTTCATAGT |
| 5-*atp*E-F | AACAAAGCTGCGGATCTCGA |
| 6-*chl*L-R | TTGATTTACCTGTGCCGCCT |
| 7-*ccsA*-6F | GAAAACTGCAGTTACCCCGC |
| 8-*rrn*5-R | GTAGAGGAACCACACCAATC |
| 9-*pet*N-F | GCAGCCCAAGCAGTATTGAC |
| 10-*rpoB*-R | GTTTCTCCCATCGGTTTCCAATTT |
