# Supplementary material for: Directed Repeats Co-occur with Few Short-Dispersed Repeats in Plastid Genome of a Spikemoss, Selaginella vardei (Selaginellaceae, Lycopodiopsida)
Source: BMC Genomics. 2019 Jun 11;20:484. doi: 10.1186/s12864-019-5843-6 (PMC6560725; doi:10.1186/s12864-019-5843-6)
Supplement: Supplementary file 10 — Table S5. Detailed dispersed short repeats in plastomes of each species. (DOCX 45 kb) [file 12864_2019_5843_MOESM10_ESM.docx]

Table S5-1 Detailed dispersed short repeats in plastome of *Huperzia serrata.*

| q_start | q_end | length | s_start | s_end | length | mismatch | q_location | s_location |
| --- | --- | --- | --- | --- | --- | --- | --- | --- |
| 2768 | 2784 | 17 | 7406 | 7422 | 17 | 0 | *rpl22* | *rps11* |
| 7451 | 7468 | 18 | 79118 | 79101 | -18 | 0 | *rps11* | *atpA* |
| 10278 | 10294 | 17 | 40757 | 40773 | 17 | 0 | *petD-petB* | *trnF-GAA-trnL-UAA* |
| 11926 | 11947 | 22 | 89620 | 89597 | -24 | 2 | *petB-psbH* | *rpoC2* |
| 22289 | 22308 | 20 | 116350 | 116331 | -20 | 0 | *trnW-CCA* | *trnN-GUU* |
| 23710 | 23726 | 17 | 78466 | 78450 | -17 | 0 | *petL-psbE* | *ycf12-trnR-UCU* |
| 30154 | 30172 | 19 | 30219 | 30201 | -19 | 0 | *ycf4-psaI* | *ycf4-psaI* |
| 30642 | 30660 | 19 | 105387 | 105405 | 19 | 0 | *psaI-accD* | *rps7-trnV-GAC* |
| 37024 | 37040 | 17 | 89012 | 88996 | -17 | 0 | *trnV-UAC* | *rpoC2* |
| 39699 | 39719 | 21 | 85693 | 85713 | 21 | 0 | *ndhJ-trnF-GAA* | *rpoC2* |
| 39997 | 40015 | 19 | 109837 | 109819 | -19 | 0 | *trnF-GAA* | *trnA-UGC* |
| 44574 | 44591 | 18 | 1329 | 1312 | -18 | 0 | *ycf3* | *rpl2* |
| 51797 | 51814 | 18 | 118683 | 118700 | 18 | 0 | *rps14* | *trnN-GUU-chlL* |
| 52430 | 52456 | 27 | 77336 | 77310 | -27 | 1 | *trnG-UCC* | *ycf12-trnR-UCU* |
| 74900 | 74917 | 18 | 5211 | 5194 | -18 | 0 | *psbK-psbI* | *rpl16-rpl14* |
| 74926 | 74961 | 36 | 74961 | 74926 | -36 | 0 | *psbK-psbI* | *psbK-psbI* |
| 77726 | 77746 | 21 | 40347 | 40366 | 20 | 1 | *ycf12-trnR-UCU* | *trnF-GAA-trnL-UAA* |
| 77773 | 77789 | 17 | 138567 | 138551 | -17 | 0 | *ycf12-trnR-UCU* | *rpl21-ndhF* |
| 84298 | 84321 | 24 | 84321 | 84298 | -24 | 0 | *atpI-rps2* | *atpI-rps2* |
| 85292 | 85315 | 24 | 85577 | 85600 | 24 | 1 | *rpoC2* | *rpoC2* |
| 85693 | 85713 | 21 | 39699 | 39719 | 21 | 0 | *rpoC2* | *ndhJ-trnF-GAA* |
| 87843 | 87878 | 36 | 87771 | 87806 | 36 | 2 | *rpoC2* | *rpoC2* |
| 97613 | 97655 | 43 | 97655 | 97613 | -43 | 1 | *petN-ycf66* | *petN-ycf66* |
| 103608 | 103640 | 33 | 103640 | 103608 | -33 | 4 | *rps7-trnV-GAC* | *rps7-trnV-GAC* |
| 103621 | 103647 | 27 | 127947 | 127972 | 26 | 3 | *rps7-trnV-GAC* | *rps15-ndhH* |
| 103987 | 104080 | 94 | 94 | 1 | -94 | 0 | *rps7-trnV-GAC* | *rps12-trnI* |
| 108722 | 108739 | 18 | 116387 | 116370 | -18 | 0 | *trnI-GAU* | *trnN-GUU* |
| 111360 | 111385 | 26 | 111479 | 111504 | 26 | 1 | *trnA-UGC-rrn23* | *trnA-UGC-rrn23* |
| 111422 | 111459 | 38 | 111607 | 111644 | 38 | 3 | *trnA-UGC-rrn23* | *trnA-UGC-rrn23* |
| 116549 | 116578 | 30 | 116595 | 116624 | 30 | 0 | *trnN-GUU-chlL* | *trnN-GUU-chlL* |
| 132665 | 132682 | 18 | 132682 | 132665 | -18 | 0 | *ndhG-ndhE* | *ndhG-ndhE* |

Table S5-2 Detailed dispersed short repeats in plastome of *Isoetes flaccida*.

| q_start | q_end | length | s_start | s_end | length | mismatch | q_location | s_location |
| --- | --- | --- | --- | --- | --- | --- | --- | --- |
| 6875 | 6894 | 20 | 109935 | 109955 | 21 | 1 | *rpl36-rps11* | *ycf1-rps15* |
| 12322 | 12365 | 44 | 12365 | 12322 | -44 | 0 | *psbT-psbB* | *psbT-psbB* |
| 13872 | 13891 | 20 | 67133 | 67114 | -20 | 1 | *psbB* | *psbI* |
| 29383 | 29422 | 40 | 29422 | 29383 | -40 | 0 | *psaI-accD* | *psaI-accD* |
| 30087 | 30115 | 29 | 30115 | 30087 | -29 | 4 | *accD* | *accD* |
| 33215 | 33236 | 22 | 73587 | 73565 | -23 | 1 | *rbcL-atpB* | *atpH-atpI* |
| 33275 | 33291 | 17 | 108651 | 108635 | -17 | 0 | *rbcL-atpB* | *ycf1* |
| 35711 | 35728 | 18 | 104015 | 103998 | -18 | 0 | *trnV-UAC* | *trnN-GUU* |
| 36429 | 36446 | 18 | 36446 | 36429 | -18 | 0 | *trnV-UAC-ndhC* | *trnV-UAC-ndhC* |
| 45433 | 45456 | 24 | 45456 | 45433 | -24 | 2 | *ycf3-psaA* | *ycf3-psaA* |
| 57698 | 57714 | 17 | 128977 | 128961 | -17 | 0 | *trnT-GGU-trnE-UUC* | *ycf2* |
| 62364 | 62383 | 20 | 123567 | 123548 | -20 | 1 | *matK-rps16* | *ndhF-chlN* |
| 67205 | 67226 | 22 | 52014 | 51993 | -22 | 0 | *trnS-GCU* | *trnS-UGA* |
| 67206 | 67229 | 24 | 42352 | 42375 | 24 | 0 | *trnS-GCU* | *trnS-GGA* |
| 68975 | 69004 | 30 | 51204 | 51176 | -29 | 1 | *trnG-UCC* | *trnG-GCC* |
| 77326 | 77342 | 17 | 108206 | 108222 | 17 | 0 | *rpoC2* | *ycf1* |
| 81780 | 81796 | 17 | 62984 | 63000 | 17 | 0 | *rpoC1* | *matK-rps16* |
| 86505 | 86522 | 18 | 66093 | 66076 | -18 | 0 | *trnC-GCA* | *trnQ-UUG* |
| 89618 | 89726 | 109 | 75425 | 75533 | 109 | 2 | *ndhB* | *rps2-rpoC2* |
| 93414 | 93451 | 38 | 43907 | 43870 | -38 | 2 | *rps7-trnV-GAC* | *ycf3* |
| 97714 | 97739 | 26 | 35705 | 35729 | 25 | 1 | *trnI-GAU* | *trnV-UAC* |
| 98733 | 98751 | 19 | 39447 | 39429 | -19 | 0 | *trnA-UGC* | *trnF-GAA* |
| 103959 | 103978 | 20 | 20717 | 20698 | -20 | 0 | *trnN-GUU* | *trnW-CCA* |
| 103998 | 104015 | 18 | 97738 | 97721 | -18 | 0 | *trnN-GUU* | *trnI-GAU* |
| 123280 | 123297 | 18 | 74455 | 74472 | 18 | 0 | *ndhF-chlN* | *atpI-rps2* |
| 129285 | 129326 | 42 | 129326 | 129285 | -42 | 2 | *ycf2* | *ycf2* |
| 129626 | 129643 | 18 | 1515 | 1498 | -18 | 0 | *ycf2* | *rpl2* |
| 131308 | 131329 | 22 | 50943 | 50964 | 22 | 1 | *ycf2* | *rps14-trnfM-CAU* |

Table S5-3 Detailed dispersed short repeats in plastome of *Selaginella uncinata*.

| q_start | q_end | length | s_start | s_end | length | mismatch | q_location | s_location |
| --- | --- | --- | --- | --- | --- | --- | --- | --- |
| 6158 | 6203 | 46 | 26015 | 25970 | -46 | 3 | *psbI* | *trnC-psbK* |
| 8406 | 8423 | 18 | 8423 | 8406 | -18 | 0 | *ycf12-atpA* | *ycf12-atpA* |
| 11484 | 11500 | 17 | 41714 | 41730 | 17 | 0 | *atpA-atpH* | *ycf2-trnF* |
| 11485 | 11501 | 17 | 41711 | 41727 | 17 | 0 | *atpA-atpH* | *ycf2-trnF* |
| 14941 | 14958 | 18 | 14958 | 14941 | -18 | 0 | *rpoC2* | *rpoC2* |
| 28374 | 28394 | 21 | 25744 | 25764 | 21 | 1 | *trnC-chlB* | *trnC* |
| 30693 | 30712 | 20 | 30712 | 30693 | -20 | 0 | *chlB-matK* | *chlB-matK* |
| 35005 | 35090 | 86 | 88502 | 88586 | 85 | 2 | *trnH* | *trnN-rps4* |
| 54612 | 54709 | 98 | 63898 | 63801 | -98 | 2 | *petA-psbJ* | *rpl20-psbB* |
| 54695 | 54711 | 17 | 4530 | 4514 | -17 | 0 | *petA-psbJ* | *rps7-psbM* |
| 58325 | 58342 | 18 | 88188 | 88205 | 18 | 0 | *trnW* | *trnN* |
| 65309 | 65329 | 21 | 72919 | 72898 | -22 | 1 | *psbB* | *rps8-rpl14* |
| 81912 | 81929 | 18 | 88244 | 88227 | -18 | 0 | *rrn16-rrn23* | *trnN* |
| 91822 | 91838 | 17 | 92191 | 92207 | 17 | 0 | *ndhF* | *ndhF* |
| 99531 | 99547 | 17 | 48270 | 48254 | -17 | 0 | *ndhI* | *atpB-rbcL* |
| 113402 | 113422 | 21 | 25744 | 25764 | 21 | 1 | *trnQ* | *trnC* |
| 116057 | 116075 | 19 | 82196 | 82178 | -19 | 0 | *trnD-trnY* | *rrn16-rrn23* |
| 120463 | 120486 | 24 | 94431 | 94408 | -24 | 1 | *psbC* | *rpl21-ccsA* |
| 126222 | 126239 | 18 | 126239 | 126222 | -18 | 0 | *psaA* | *psaA* |

Table S5-4 Detailed dispersed short repeats in plastome of *S. moellendorffii*.

| q_start | q_end | length | s_start | s_end | length | mismatch | q_location | s_location |
| --- | --- | --- | --- | --- | --- | --- | --- | --- |
| 7664 | 7685 | 22 | 28762 | 28741 | -22 | 1 | *trnC* | *trnQ* |
| 17320 | 17345 | 26 | 90940 | 90916 | -25 | 2 | *rpoC2* | *rrn5-trnR* |
| 19177 | 19196 | 20 | 4972 | 4953 | -20 | 0 | *rpoC2-atpI* | *ndhB-psbM* |
| 30714 | 30735 | 22 | 4762 | 4784 | 23 | 1 | *chlB-matK* | *ndhB-psbM* |
| 30715 | 30739 | 25 | 4759 | 4785 | 27 | 2 | *chlB-matK* | *ndhB-psbM* |
| 42960 | 43003 | 44 | 43003 | 42960 | -44 | 2 | *trnD-trnY* | *trnD-trnY* |
| 54375 | 54424 | 50 | 54424 | 54375 | -50 | 0 | *accD* | *accD* |
| 59643 | 59659 | 17 | 93831 | 93815 | -17 | 0 | *petA-psbJ* | *trnN-rps4* |
| 63725 | 63750 | 26 | 6245 | 6270 | 26 | 2 | *trnW-psaJ* | *psbM-petN* |
| 65911 | 65938 | 28 | 65880 | 65907 | 28 | 1 | *rpl20-clpP* | *rpl20-clpP* |
| 90669 | 90688 | 20 | 90640 | 90621 | -20 | 0 | *rrn4.5-rrn5* | *rrn4.5-rrn5* |
| 91729 | 91747 | 19 | 86464 | 86482 | 19 | 0 | *trnR-trnN* | *rrn16-rrn23* |
| 93447 | 93493 | 47 | 36147 | 36193 | 47 | 1 | *trnN-rps4* | *trnH* |
| 95235 | 95253 | 19 | 95180 | 95162 | -19 | 0 | *rps4-ndhF* | *rps4-ndhF* |
| 99187 | 99213 | 27 | 99160 | 99186 | 27 | 1 | *trnL-ccsA* | *trnL-ccsA* |
| 117634 | 117657 | 24 | 77681 | 77704 | 24 | 0 | *chlL-trnF* | *rps8-rpl14* |
| 129587 | 129603 | 17 | 130660 | 130676 | 17 | 0 | *ycf3* | *ycf3* |
| 129974 | 130015 | 42 | 130015 | 129974 | -42 | 0 | *ycf3* | *ycf3* |

Table S5-5 Detailed dispersed short repeats in plastome of *S. vardei*.

| q_start | q_end | length | s_start | s_end | length | mismatch | q_location | s_location |
| --- | --- | --- | --- | --- | --- | --- | --- | --- |
| 17397 | 17412 | 16 | 42152 | 42137 | -16 | 0 | *ycf12-psbI* | *psaA* |
| 25341 | 25356 | 16 | 58272 | 58257 | -16 | 0 | *ycf2* | *psbM-petN* |
| 25733 | 25748 | 16 | 33858 | 33843 | -16 | 0 | *ycf2* | *psbD* |
| 57908 | 57925 | 18 | 57974 | 57991 | 18 | 0 | *psbM-petN* | *psbM-petN* |
| 73582 | 73597 | 16 | 75269 | 75284 | 16 | 0 | *psbB* | *psbB-clpP* |
| 88082 | 88098 | 17 | 102166 | 102182 | 17 | 0 | *rbcL* | *ycf1-psaC* |

Table S5-6 Detailed dispersed short repeats in plastome of *S. indica*.

| q_start | q_end | length | s_start | s_end | length | mismatch | q_location | s_location |
| --- | --- | --- | --- | --- | --- | --- | --- | --- |
| 16906 | 16929 | 24 | 64579 | 64556 | -24 | 1 | *ycf12*-*psbI* | *rpl16* |
| 17404 | 17419 | 16 | 42074 | 42059 | -16 | 0 | *ycf12*-*psbI* | *psaA* |
| 17492 | 17508 | 17 | 60223 | 60207 | -17 | 0 | *ycf12*-*psbI* | *trnC*-*rpl2* |
| 46525 | 46544 | 20 | 79761 | 79780 | 20 | 0 | *trnN* | *trnW* |
| 88962 | 88978 | 17 | 103023 | 103039 | 17 | 0 | *rbcL* | *ycf1*-*psaC* |

Table S5-7 Detailed dispersed short repeats in plastome of *S. tamariscina*.

| q_start | q_end | length | s_start | s_end | length | mismatch | q_location | s_location |
| --- | --- | --- | --- | --- | --- | --- | --- | --- |
| 2335 | 2353 | 19 | 78683 | 78701 | 19 | 0 | *rps7-psbM* | *psaJ-clpP* |
| 3753 | 3768 | 16 | 47342 | 47327 | -16 | 0 | *psbM-petN* | *ccsA-rps4* |
| 35473 | 35488 | 16 | 15461 | 15476 | 16 | 0 | *chlN* | *rpoC2* |
| 54522 | 54542 | 21 | 42317 | 42336 | 20 | 1 | *rrn23* | *psaC-ccsA* |
| 62690 | 62714 | 25 | 47961 | 47985 | 25 | 2 | *rps3-rpl14* | *rps4* |
| 76625 | 76644 | 20 | 49437 | 49418 | -20 | 0 | *trnW* | *trnN* |
| 84266 | 84282 | 17 | 72447 | 72463 | 17 | 0 | *psbI* | *psbB* |
| 84479 | 84499 | 21 | 82978 | 82959 | -20 | 1 | *psbI-psbK* | *petA-ycf12* |
| 86018 | 86033 | 16 | 31188 | 31203 | 16 | 0 | *chlB* | *atpE-ndhC* |
| 100828 | 100845 | 18 | 82604 | 82587 | -18 | 0 | *trnE-psbD* | *petA-ycf12* |
| 109671 | 109686 | 16 | 100495 | 100510 | 16 | 0 | *psaA* | *trnE-psbD* |

Table S5-8 Detailed dispersed short repeats in plastome of *S. kraussiana*.

| q_start | q_end | length | s_start | s_end | length | mismatch | q_location | s_location |
| --- | --- | --- | --- | --- | --- | --- | --- | --- |
| 62825 | 62900 | 76 | 62773 | 62848 | 76 | 4 | *rpl2-ndhF* | *rpl2-ndhF* |
| 38035 | 38073 | 39 | 37996 | 38034 | 39 | 0 | *rps18-clpP* | *rps18-clpP* |
| 12592 | 12608 | 17 | 1623 | 1607 | -17 | 0 | *ycf1* | *ccsA* |
| 91795 | 91810 | 16 | 4775 | 4760 | -16 | 0 | *psbA* | *psaC-ndhE* |
| 86289 | 86304 | 16 | 6727 | 6712 | -16 | 0 | *ycf2* | *ndhI* |
| 33150 | 33165 | 16 | 20580 | 20565 | -16 | 0 | *petA-psbJ* | *ndhJ* |

Table S5-9 Detailed dispersed short repeats in plastome of *S. lepidophylla*.

| q_start | q_end | length | s_start | s_end | length | mismatch | q_location | s_location |
| --- | --- | --- | --- | --- | --- | --- | --- | --- |
| 92902 | 92919 | 18 | 87227 | 87210 | -18 | 0 | *trnE-UUC-psbD* | *rps7-rpl21* |
| 34119 | 34135 | 17 | 35432 | 35448 | 17 | 0 | *trnF-GAA* | *trnF-GAA-chlL* |
| 79221 | 79240 | 20 | 23449 | 23467 | 19 | 1 | *psbM-rps7* | *petA* |
| 95135 | 95150 | 16 | 29801 | 29786 | -16 | 0 | *psbD* | *rbcL* |
| 59233 | 59248 | 16 | 41553 | 41538 | -16 | 0 | *atpA* | *ycf1* |
| 68105 | 68120 | 16 | 55580 | 55595 | 16 | 0 | *rpoC2* | *chlB-psbK* |
| 34140 | 34156 | 17 | 35356 | 35372 | 17 | 0 | *trnF-GAA-chlL* | *trnF-GAA-chlL* (16 copies) |
