# Supplementary material for: Directed Repeats Co-occur with Few Short-Dispersed Repeats in Plastid Genome of a Spikemoss, Selaginella vardei (Selaginellaceae, Lycopodiopsida)
Source: BMC Genomics. 2019 Jun 11;20:484. doi: 10.1186/s12864-019-5843-6 (PMC6560725; doi:10.1186/s12864-019-5843-6)
Supplement: Supplementary file 11 — Table S6. Species selected in the phylogenetic analyses. (DOCX 15 kb) [file 12864_2019_5843_MOESM11_ESM.docx]

Table S6 Species selected in the phylogenetic analyses

| **Species** | **GenBank No.** |
| --- | --- |
| *Amborella trichopoda* | NC 005086 |
| *Azolla filiculoides* | MF177094 |
| *Dendrolycopodium obscurum* | MH549637 |
| *Diphasiastrum digitatum* | MH549638 |
| *Isoetes flaccida* | NC 014675 |
| *Isoetes malinverniana* | MH549640 |
| *Isoetes nuttallii* | NC038073 |
| *Huperzia javanica* | KY609860 |
| *Huperzia lucidula* | AY660566 |
| *Huperzia serrata* | NC 033874 |
| *Lepisorus clathratus* | NC035739 |
| *Lycopodium clavatum* | MH549642 |
| *Osmundastrum cinnamomeum* | KF225592 |
| *Ophioglossum californicum* | NC 020147 |
| *Physcomitrella patens* | AP005672 |
| *Pinus thunbergii* | NC 001631 |
| *Salvinia cucullata* | MF177095 |
| ***Selaginella indica*** | ***MK156801** |
| *Selaginella kraussiana* | MH549643 |
| *Selaginella lepidophylla* | MK089531 |
| *Selaginella moellendorffii* | FJ755183 |
| *Selaginella uncinata* | AB197035 |
| *Selaginella tamariscina* | - |
| ***Selaginella vardei*** | ***MG272482** |

- GenBank number unavailable

* newly sequenced plastome
